# Multi-agent deep reinforcement learning-based robotic arm assembly research

**DOI:** 10.1371/journal.pone.0311550

**Published:** 2025-02-18

**Authors:** Guohua Cao, Jimeng Bai

**Affiliations:** School of Mechanical and Electrical Engineering, Changchun University of Science and Technology, Changchun, China; North-West University Potchefstroom Campus: North-West University, SOUTH AFRICA

## Abstract

Due to the complexity and variability of application scenarios and the increasing demands for assembly, single-agent algorithms often face challenges in convergence and exhibit poor performance in robotic arm assembly processes. To address these issues, this paper proposes a method that employs a multi-agent reinforcement learning algorithm for the shaft-hole assembly of robotic arms, with a specific focus on square shaft-hole assemblies. First, we analyze the stages of hole-seeking, alignment, and insertion in the shaft-hole assembly process, based on a comprehensive study of the interactions between shafts and holes. Next, a reward function is designed by integrating the decoupled multi-agent deterministic deep deterministic policy gradient (DMDDPG) algorithm. Finally, a simulation environment is created in Gazebo, using circular and square shaft-holes as experimental subjects to model the robotic arm’s shaft-hole assembly. The simulation results indicate that the proposed algorithm, which models the first three joints and the last three joints of the robotic arm as multi-agents, demonstrates not only enhanced adaptability but also faster and more stable convergence.

## 1.Introduction

Advances in robotic technology have led to increasingly widespread applications of robots in the field of industrial automation. Shaft-hole assembly, a critical task in mechanical manufacturing, demands highly precise and efficient control methods to ensure quality and productivity. This task is inherently multivariable, requiring simultaneous control of both the position and orientation of the robotic arm. Traditional control methods for shaft-hole assembly, such as model predictive control [1] or force/position hybrid control [2], rely on the prior determination of model parameters and control settings. However, due to the nonlinearity and complexity of robotic arms, these methods often fall short of achieving the desired control performance in complex assembly processes.

In recent years, deep reinforcement learning (DRL) [3] has garnered significant attention for its potential in robotic arm control. Unlike conventional control strategies, DRL does not necessitate explicit model construction or control strategy design. Instead, it adaptively adjusts control strategies, accommodating various environmental conditions and task requirements, thereby offering superior adaptability and generalization capabilities. This enables the learning of more complex control strategies, ultimately leading to more efficient and precise shaft-hole assembly.

DRL represents an emerging intelligent control approach, fundamentally based on learning from the current state and predefined goals through trial-and-error interactions with the environment. The system autonomously refines decisions and executes optimal actions. DRL has already found extensive applications in the field of robotic control [4], achieving significant outcomes in areas such as robotic grasping [5,6], path planning [7], polishing [8], and welding [9]. In the context of shaft-hole assembly, the complexity and variability of the shaft-hole shapes and sizes make the motion control of robotic arms a challenging task. Single-agent DRL algorithms can effectively handle relatively simple assembly tasks, such as the assembly of fully symmetric circular shaft-holes [10]. However, for more complex shaft-hole assemblies, higher precision and efficiency are required, necessitating more accurate control of both position and orientation.

Multi-agent deep reinforcement learning (MADRL) is a method that enables collaboration among multiple agents, extending single-agent reinforcement learning by forming a group of agents to collaboratively complete tasks. Its application in robotic arm control primarily involves cooperative control and task allocation. Each agent within the group must not only ensure internal consistency but also consider cooperation with other agents, thereby advancing the overall system towards its goal while completing individual tasks. The collaboration among multiple agents simplifies complex tasks, enhancing control precision and efficiency. However, the increase in the number of agents can lead to challenges such as data dimensionality explosion, communication difficulties, and convergence issues [11]. Additionally, the strong coupling between the joints and links of the robotic arm introduces further complexities in control.

To address these issues, this paper explores the application of multi-agent deep reinforcement learning algorithms in the motion control of a single robotic arm. Focusing on complex shaft-hole assembly, a shaft-hole assembly system based on the DMDDPG algorithm is designed, targeting position and orientation as controlled variables to effectively improve assembly efficiency and generalization in different assembly environments. A global reward function is designed for the task objectives and shaft-hole assembly process, and to reduce coupling between agents, a local reward function is independently designed for each agent. Consequently, the proposed algorithm can leverage both global and local rewards to guide the exploration and optimization of assembly strategies. Comparative simulation experiments with other reinforcement learning algorithms show that the proposed algorithm excels in efficiency and reliability.

## 2. Premilaries

### 2.1 Multi-agent deep reinforcement learning algorithm

A classic multi-agent algorithm is the MDDPG, which extends the DDPG algorithm to multi-agent environments and introduces the centralized training-decentralized execution framework. Each agent in this framework is a complete DDPG model. During training, each agent’s Critic network uses the actions and states of all agents, while the Actor network updates its policy based solely on its observed state. Consequently, each agent’s Critic network fits the global value function rather than an individual value function, allowing each agent’s policy to be updated towards the optimal global value function.

In multi-agent deep reinforcement learning, the collaborative exploration process is described by a Markov game, also known as a stochastic game. This concept encompasses two key ideas: first, the multi-agent system follows Markov properties; second, the game describes the relationships among the agents. The multi-agent Markov game process is defined as a tuple (*N*,*S*,*A*,*T*,*R*,*γ*), where N represents the number of agents; *S* = (*x*,*o*_1_,*o*_2_,⋯*o*_*N*_) is the set of joint states; *o*_*i*_ is the observed state of agent *i*, and *x* is other environmental information; *A* = (*a*_1_,*a*_2_,⋯,*a*_*N*_) is the set of joint actions; *a*_*i*_ represents the actions of agent *i*; *T*∈[0,1] is the state transition probability of *S*→*A*→*S*′; *R* = (*r*_1_,*r*_2_,⋯,*r*_*N*_), *r*_*i*_ represents the reward received by agent *i* for performing action *a*_*i*_, which leads to outcome *o*→*o*′. *γ* denotes the discount factor for the cumulative reward.

In a multi-agent system, changes in the environmental state result from the collective actions of all agents. At time t, each agent combines its observed state $o_{i}^{t}$ and executes *π*_*i*_(*a*_*i*_|*o*_*i*_) joint action $A^{t}=(a_{i}^{t},a_{2}^{t},\cdots a_{N}^{t})$, causing a state transition in the environment and receiving an expected reward $r_{i}^{t}$ for the action taken.


$$r_{i}^{t}=E[r_{i}^{t+1}|S^{t}=o_{i},A_{i}^{t}=a_{i},\pi_{i}]$$ (1)

The Critic network of each agent takes the same input parameters: the observed environment states *S* of all agents, the actions *A* of the agents, and the corresponding Critic network parameters $\theta_{i}^{Q}$, producing the global value function as output:

$$Q^{H}(S,a_{1},\ldots ,a_{N})=E[R+\gamma Q^{H}(S^{\prime },a_{1}^{\prime },\ldots ,a_{N}^{\prime })]$$ (2)

where $H=\prod_{i\in N}\pi_{i}(a_{i}|o_{i})$ represents the joint policy of all agents.

Each agent’s Actor network parameters $\theta_{i}^{\pi }$ are updated according to the value function through gradient descent:

$$\nabla_{\theta_{i}^{\pi }}J(\theta_{i}^{\pi })=E_{S,A∼D}[\nabla_{\theta_{i}^{\pi }}\log\pi_{i}(a_{i}|o_{i})Q_{i}^{\pi }(S,a_{1},\cdots a_{N})]$$ (3)

where *D* represents the experience replay buffer, with each element being a tuple (*S*,*A*,*R*,*S*′), containing the updated joint state *S*′.


$$L(\theta_{i}^{Q})=E_{S,A∼D}[{\left(Q_{i}^{H}(S,a_{1},\cdots a_{N})-y\right)}^{2}]$$ (4)

The Critic network parameters are updated using backpropagation, with the loss function defined as:

$$y=r_{i}+\gamma Q_{i}^{H^{\prime }}(S^{\prime },a_{1}^{\prime },\cdots a_{N}^{\prime })|a_{i}^{\prime }=\pi_{i}^{\prime }(a_{i}|o_{i})$$ (5)

### 2.2 Current research on robotic arm assembly control technologies

The development of modern control theory has gradually positioned active compliance control as a primary research focus [12,13]. This approach enables robots to execute corresponding control strategies based on their perception of the environment to accomplish assembly tasks, without the need for dedicated compliance mechanisms, thereby offering enhanced versatility. Hogan et al. introduced the fundamental principles of impedance control, establishing a mapping between the robot’s position and contact force [14]. This work laid the groundwork for subsequent active compliance research, leading to numerous improved control strategies adapted to various environments. For instance, Wang et al. designed a joint impedance controller using sliding mode control, which effectively enhances the flexibility of the joints, allowing the robot to execute shaft-hole assembly tasks with greater precision [15]. This controller also addresses model uncertainties and reduces impact forces, thereby improving the robot’s disturbance rejection capability. Wu et al. proposed a control method for flexible connectors based on an event-switching strategy, which enables a smooth transition from adaptive control to hybrid impedance control [16]. This approach enhances control performance in both constrained and unconstrained spaces, ensuring smooth completion of connector assembly tasks.

The application and advancement of computer technology have significantly improved the computational power of robotic control systems, enabling breakthroughs in robotic arm assembly methods by integrating artificial intelligence with traditional control algorithms [17]. Two primary approaches have emerged: image-based visual perception and force-sensor-based tactile perception. For example, Song et al. simulated human visual sensitivity to key information and introduced an information filtering mechanism to improve the accuracy of feature recognition and classification in components [18]. Cong et al. utilized machine vision to correct assembly postures, increasing the success rate in assembling irregularly shaped parts [19]. Luo et al. designed an assembly prediction network by integrating multi-view perception of missing features with deep reinforcement learning, significantly enhancing assembly efficiency and stability [20]. Ortega-Aranda et al. collected contact state information during robotic operations and trained a dual-arm robot using a neural network-based fuzzy architecture to achieve human-like performance [21]. Although impedance model-based control strategies perform well in assembly tasks, they are limited by the need for an accurate model of the shaft-hole contact forces and precise identification of dynamic parameters, which may change due to wear and fatigue during actual operations, thereby impacting assembly outcomes.

Compared to traditional control strategies, deep reinforcement learning does not require explicit model construction or control strategy design [22]. It can adaptively adjust control strategies to suit different environments and task requirements, offering superior adaptability and generalization capabilities. This enables the learning of more complex control strategies, leading to more efficient and precise shaft-hole assembly. Inoue et al. demonstrated the difficulty of obtaining accurate models in complex shaft-hole assembly and proposed a reinforcement learning algorithm tailored to shaft-hole tasks [23]. Leyendecker et al. validated the effectiveness of deep reinforcement learning assembly strategies in uncertain environments through simulations [24]. Ding et al. divided the shaft-hole assembly process into two stages—hole searching and insertion—and developed a self-learning assembly algorithm based on DQN [25]. In shaft-hole assembly, the complex and variable shapes and sizes of shaft-holes make the motion control of robotic arms particularly challenging. While single-agent deep reinforcement learning algorithms can handle relatively simple tasks, such as assembling fully symmetric circular shaft-holes, achieving higher precision and efficiency in complex shaft-hole assembly requires more accurate control of both position and orientation.

### 2.3 Current research on multi-agent reinforcement learning

Multi-agent deep reinforcement learning (MADRL) involves collaborative learning among multiple agents, expanding on single-agent reinforcement learning by forming a collective of agents to cooperatively accomplish tasks. In the context of robotic arm control, MADRL focuses on collaborative control and task allocation, where each agent not only ensures the consistency of its own capabilities but also cooperates with others to achieve the overall system’s objectives [26]. Foerster et al. introduced the reinforced inter-agent learning and differentiable inter-agent learning algorithms, which employ neural networks to approximate value functions, facilitating inter-agent communication and reducing model complexity through parameter sharing [27]. Sukhbaatar et al. proposed the communication network algorithm, enabling real-time, rapid information exchange among agents [28]. Lowe et al. developed the multi-agent deep deterministic policy gradient algorithm, which extends the DDPG framework by incorporating a centralized training with decentralized execution approach, significantly reducing learning costs and establishing a paradigm for most cooperative methods [29]. He et al. proposed an improved DDPG algorithm that treats the joints of a robotic arm as decision-making agents within a multi-agent system, enabling motion planning for target capture [30]. This approach demonstrated superior solution speed and robustness compared to traditional algorithms. While the collaboration among multiple agents simplifies complex tasks and enhances control precision and efficiency in robotic arms, it also introduces challenges such as data dimensionality explosion, communication difficulties, and convergence issues. Additionally, the strong coupling between robotic arm joints further complicates control efforts [31].

## 3. Robotic arm shaft-hole assembly using deep reinforcement learning algorithm

### 3.1 Analysis of the shaft-hole assembly task

The shaft-hole assembly process can be divided into three main stages: hole-seeking, alignment, and insertion. The hole-seeking stage involves moving the shaft from its initial position towards the centerline of the hole, reaching the vicinity of the assembly hole. The alignment stage involves adjusting the position and orientation of the shaft relative to the hole to meet assembly requirements. The insertion stage involves inserting the shaft to the specified depth after achieving the required alignment. Among these stages, the alignment stage is the most critical and complex, as it involves various contact states such as point contact, surface contact, and line contact when there is a mismatch in position and orientation. This stage is crucial for the successful completion of the assembly task.

**Fig 1:**
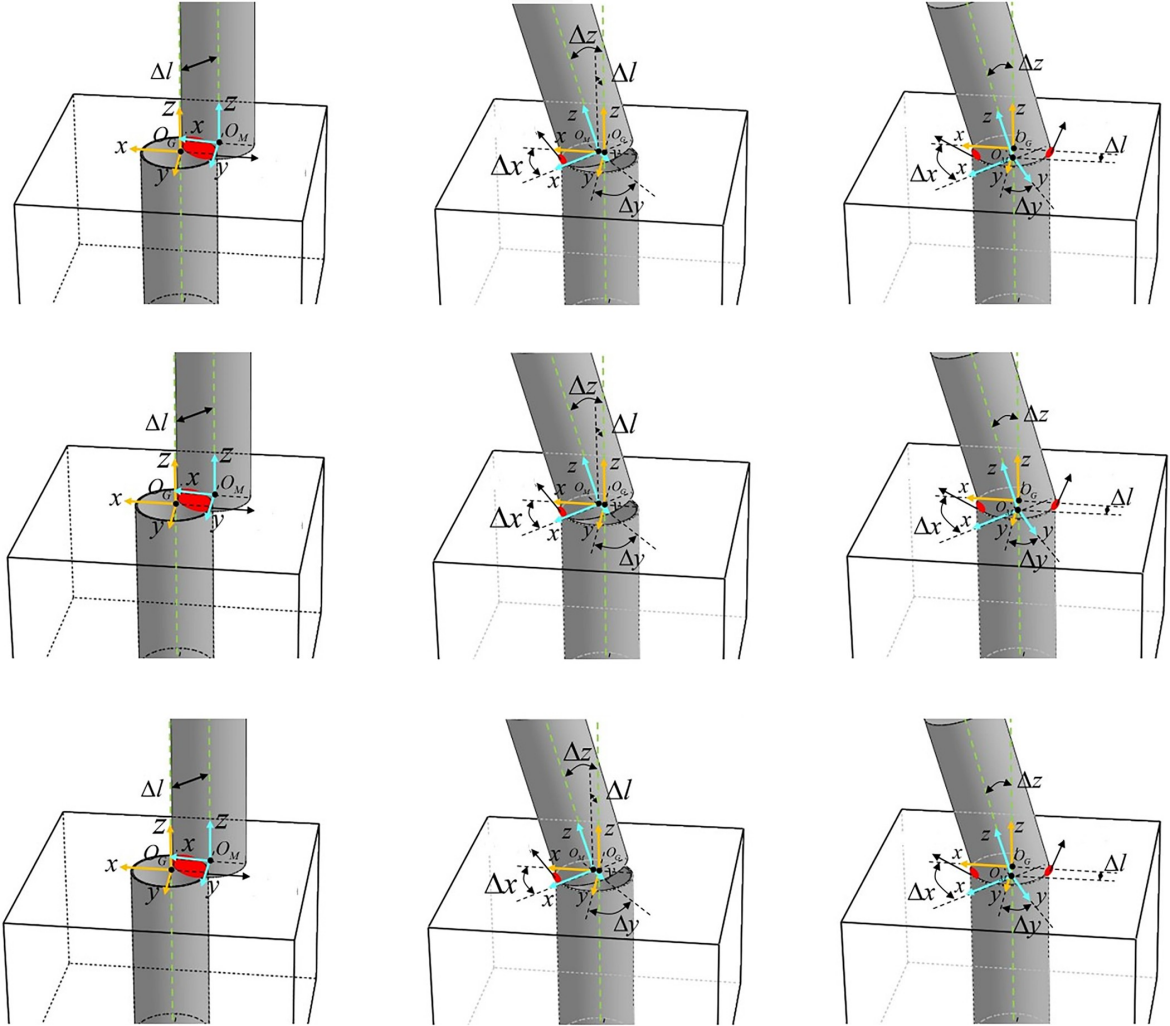
The contact state in circular shaft-hole assembly. a) Surface contact (b) Point contact (c) Multiple point contact.

This study investigates the impact of various contact states during the alignment phase in both circular and square shaft-hole assemblies, with a particular focus on comparing the differences in pose control between the two. In the circular shaft-hole assembly, a coordinate system *O*_*G*_ is established with the center of the contact surface as the origin and the hole’s central axis as the *z*-axis. Similarly, a coordinate system *O*_*M*_ is established with the shaft’s axis as the *z*-axis. The deviations in the *x*, *y*, and *z* axes between coordinate systems *O*_*G*_ and *O*_*M*_ are defined as Δ*x*, Δ*y*, and Δ*z*, respectively, while Δ*l* represents the distance between the origins of the two coordinate systems. During the alignment phase, several typical contact states may occur, as illustrated in **Fig 1**.

In the surface contact state shown in **Fig 1A**, the orientation of the shaft and hole is fully aligned, but there is a positional deviation, i.e., (Δ*x*, Δ*y*, Δ*z*) = 0, Δ*l* ≠ 0. By adjusting the position of the shaft to align it directly above the hole, positional matching can be achieved, allowing the assembly process to proceed to the insertion phase. However, in circular shaft-hole assemblies, point contact, as shown in **Fig 1B**, is more likely to occur. In this scenario, the positional deviation Δ*l* is nearly zero, but there remains a significant orientation mismatch. Due to the perfect symmetry of the circular shape, the x and y axes of the shaft-hole can rotate around the z-axis to satisfy the right-hand rule in any direction, meaning that alignment can be achieved by simply aligning the z-axes of the two coordinate systems. If the orientation is not promptly adjusted to achieve Δ*z* = 0 during the point contact state, continued insertion along the shaft axis may lead to a multiple point contact state as illustrated in **Fig 1C**. At this stage, the shaft’s contact surface origin is already within the hole, and the shaft collides with the hole wall, making further insertion unsuitable.

**Fig 2:**
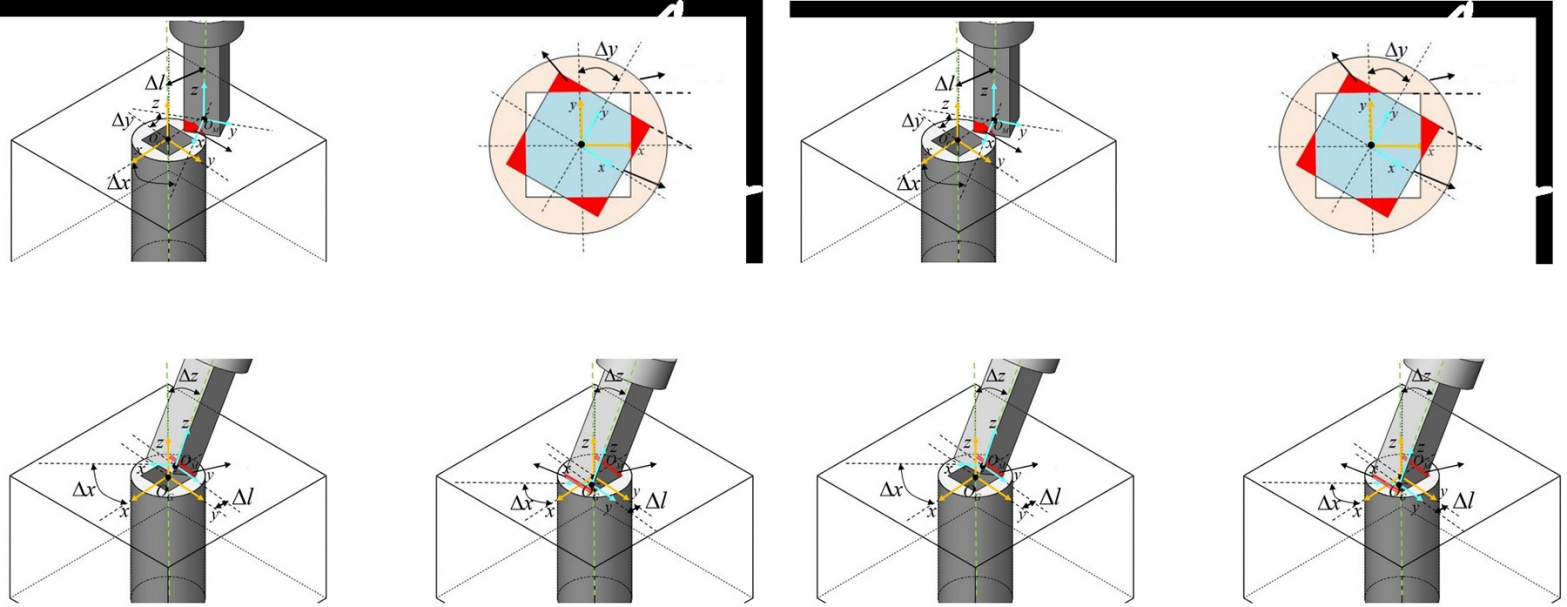
Contact states in square shaft-hole assembly. (a) Surface contact (b) Multiple surface contact (c) Line contact (d) Multiple line contact.

In the square shaft-hole assembly, the coordinate system is similarly established with the center of the contact surface as the origin and the *z*-axis defined by the hole’s central axis and the shaft’s axis. However, the *x* and *y* axes no longer have arbitrary definitions; for consistency, the directions perpendicular to the edges are defined as the *x* and *y* axes. The definitions of positional and orientation deviations are the same as in the circular shaft-hole assembly. Several contact states that may occur during the alignment phase in square shaft-hole assemblies are shown in **Fig 2**.

In **Fig 2A**, the surface contact state shows that only the z-axis of the shaft and hole is matched, but position deviation still exists. As the shaft moves to the centerline of the hole to eliminate position deviation Δ*l*, the *x* and *y* axis deviations cannot be ignored as in circular shaft-hole assembly, leading to the multiple surface contact state shown in **Fig 2B**. At this point, further rotation around the *z*-axis is required to Δ*y* = 0 proceed with the insertion task. **Fig 2C** illustrates the line contact state Δ*y* = 0,Δ*l*→0, where the other two axes are not yet matched, and if not corrected promptly, will result in multiple line contact shown in **Fig 2D**, causing scraping against the hole wall. There are also more complex contact states that cannot meet the assembly requirements for square shafts and holes.

In summary, for square shaft-hole assembly, the contact surface center of the assembly shaft must be aligned with the centerline of the hole, denoted as Δ*l* = 0. Regarding orientation, for the non-symmetrical square shaft-hole assembly, the next insertion stage can only proceed when the orientations along all three axes are fully matched, denoted as (Δ*x*,Δ*y*,Δ*z*) = 0.

Square shaft-hole assembly demands significantly higher precision than circular shaft-hole assembly, necessitating the development of specialized assembly strategies that can also accommodate the requirements of circular shaft-hole assemblies. For circular shaft-hole assembly tasks, various mature intelligent control algorithms, such as DQN and DDPG, are effective in solving these problems. However, their control strategies are not well-suited to the stringent orientation matching required for square shaft-hole assemblies.

Based on the previous analysis, the primary distinction between these two types of assembly tasks lies in the control of orientation. To address this challenge, a strategy that independently controls position and orientation is proposed to reduce the difficulty of square shaft-hole assembly tasks. This strategy introduces the MADDPG algorithm to coordinate the control of both position and orientation. However, this algorithm is typically used for collaborative control among independent entities. In the case of serial robotic arms, where the joints exhibit a certain degree of coupling, the collaborative capabilities of MADDPG are somewhat limited, which hampers its effectiveness in precise assembly tasks. To overcome these limitations, the DE-MADDPG algorithm is introduced. By incorporating a decoupling module, this algorithm reduces the coupling between position and orientation control, thereby improving the overall assembly performance.

### 3.2 Robotic arm agent configuration analysis

The assembly task focuses on the robotic arm, with the Kawasaki BA006N model selected as the control object. This industrial robot is composed of interconnected rigid links through six rotational joints. Studying the arm’s motion states involves examining the relative motion relationships between each link, necessitating the establishment of coordinate systems for each joint in the Cartesian coordinate system. The DH model is commonly used to describe changes between joint coordinate systems, categorized into Standard DH (SDH) and Modified DH (MDH). The key distinction lies in the position where the reference coordinate is established: SDH establishes the *i*-coordinate system at the end of the *i*−1 link, while MDH establishes it at the *i* joint. For a more intuitive and concise description of the robotic arm configuration, this study adopts the MDH model for modeling, requiring only four parameters to represent transformations between adjacent joint coordinate systems.

In this model: The link length (*a*) is the length of the common perpendicular line between adjacent joint axes. The link twist angle (*α*) is the angle formed between adjacent joints, with the positive direction determined by the right-hand rule. The link offset (*d*) is the distance between the projections of the common perpendicular lines of the previous and current joint axes onto the current axis. The joint angle (*θ*) is the rotation angle of two links about their common axis.

The *i* joint’s coordinate system can be transformed from the *i*−1 joint’s coordinate system by rotating around the *x*-axis by angle *α*_*i*−1_, translating along the x-axis by distance *α*_*i*−1_, rotating around the *z*-axis by angle *θ*_*i*_, and finally translating along the *z*-axis by distance *d*_*i*_. This transformation between adjacent coordinate systems is expressed as:

$$\begin{array}{l} T_{i-1}^{i}=Rot(\alpha_{i-1})Trans(a_{i-1})Rot(\theta_{i})Trans(d_{i})\\ =\left[\begin{array}{cccc} c\theta_{i} & -s\theta_{i} & 0 & a_{i-1}\\ s\theta_{i}c\alpha_{i-1} & c\theta_{i}c\alpha_{i-1} & -s\alpha_{i-1} & -d_{i}s\alpha_{i-1}\\ s\theta_{i}s\alpha_{i-1} & c\theta_{i}s\alpha_{i-1} & c\alpha_{i-1} & d_{i}c\alpha_{i-1}\\ 0 & 0 & 0 & 1 \end{array}\right] \end{array}$$ (6)

where *s* denotes sine, *c* denotes cosine, $T_{i-1}^{i}$ is the homogeneous transformation matrix of the coordinate system, and (*α*,*a*,*θ*,*d*) represents the DH parameters of the transformation matrix. Based on the relationships between joints and various data, joint coordinate systems are established using MDH, with DH parameters for each joint as shown in **Table 1**, forming the initial configuration of the robotic arm.

**Table 1:** DH parameters of BA006N robotic arm.

Link Numbering	*α*	a	*θ*	d
1	0	0	*θ*_1_+*π*/2	0.43
2	−*π*/2	0.165	*θ*_2_−*π*/2	0
3	0	0.55	*θ*_3_	0
4	−*π*/2	0.21	*θ*_4_	0.865
5	*π*/2	0	*θ*_5_	0
6	−*π*/2	0	*θ*_6_	0.115

Multiplying all joint transformation matrices yields the homogeneous transformation matrix between the base coordinate system and the end-effector coordinate system. Since only the joint angle is involved in the coordinate transformation parameters, reflecting the mapping relationship between the end-effector’s pose in Cartesian space and the robotic arm joint rotation angles, it is defined as the kinematic equation of the robotic arm *F*(*θ*):

$$F(\theta )=T_{0}^{1}T_{1}^{2}\cdots T_{5}^{6}=[\begin{array}{cc} R & P\\ 0 & 1 \end{array}]$$ (7)

where *P* represents the position matrix of the end effector in the base coordinate system of the robotic arm, denoted as *P* = [*p*_*x*_,*p*_*y*_,*p*_*z*_]^*T*^, *R* represents the orientation rotation matrix of the end effector in 3×3 Cartesian space, represented by a matrix composed of a unit vector along the principal axis direction (*x*,*y*,*z*), denoted as [*n o a*].


$$R=[n\;o\;a]=[\begin{array}{ccc} n_{x} & o_{x} & a_{x}\\ n_{y} & o_{y} & a_{y}\\ n_{z} & o_{z} & a_{z} \end{array}]$$ (8)

In addition to rotation matrices, other methods for describing orientation include Euler angles and quaternions. Euler angles require only three angles to describe the rotation between axes, but they impose strict requirements on rotation order and may encounter gimbal lock issues in continuous poses. Quaternions define orientation rotation as rotating by a specified angle around a specific rotation axis, effectively avoiding gimbal lock. They require only a four-dimensional vector composed of one scalar and a three-dimensional vector to represent, reducing data storage compared to rotation matrix operations, improving computational efficiency, and enabling smooth description of orientation changes in continuous poses. The expression for quaternions is:

q=ω+ai+bj+ck (9)

where *ω* represents the scalar real part, while *i*, *j*, and *k* constitute the imaginary three-dimensional vector, satisfying *i*^2^ = *j*^2^ = *k*^2^ = *i*⋅*j*⋅*k* = −1, where *ω*, *a*, *b*, and *c* are all real numbers. The formula for computing the quaternion norm is:

$$|q|=\sqrt{\omega^{2}+a^{2}+b^{2}+c^{2}}$$ (10)

When the quaternion norm equals 1, it is a unit quaternion. Unless otherwise specified, all quaternions described in this paper are unit quaternions. To explicitly describe the rotational relationship of the orientation, they are denoted as follows:

$$q=[\begin{array}{cc} \cos(\frac{\theta }{2}) & \sin(\frac{\theta }{2})\vec{r} \end{array}]=[\begin{array}{cccc} \cos(\frac{\theta }{2}) & \sin(\frac{\theta }{2})i & \sin(\frac{\theta }{2})j & \sin(\frac{\theta }{2}) \end{array}k]$$ (11)

where *θ* denotes the angle of rotation, $\vec{r}$ represents the rotation axis, and $\left[\begin{array}{ccc} i & j & k \end{array}\right]$ represents the direction vector of the rotation axis.

Rotation matrices and quaternions are used to describe orientation in different ways and can be mutually converted. The rotation matrix *R* obtained from the robotic arm kinematics is transformed into a quaternion *q* to describe the orientation, with the transformation formula given by:

$$\omega =\frac{1}{2}\sqrt{1+n_{x}+o_{y}+a_{z}}$$ (12)

$$\vec{r}=\frac{1}{2}[\begin{array}{c} sign(o_{z}-a_{y})\sqrt{1+n_{x}-o_{y}-a_{z}}\\ sign(a_{x}-n_{z})\sqrt{1-n_{x}+o_{y}-a_{z}}\\ sign(n_{y}-o_{x})\sqrt{1-n_{x}-o_{y}+a_{z}} \end{array}]$$ (13)

With the forward kinematics as shown in Eq (7), the position and orientation of the assembly axis end can be obtained, and based on the approach of separating position and orientation control proposed in the previous section, a design of intelligent agents is conducted for the Kawasaki BA006N robotic arm. The BA006N robotic arm adheres to the standard Pieper criteria, where three adjacent joint axes are either intersecting at one point or parallel along three axes. For this type of robotic arm configuration, the last three joints constitute the wrist of the arm, primarily controlling the end effector orientation *R*, while the first three joints mainly control the wrist position, thereby affecting the variation of the end position *P*.

**Fig 3:**
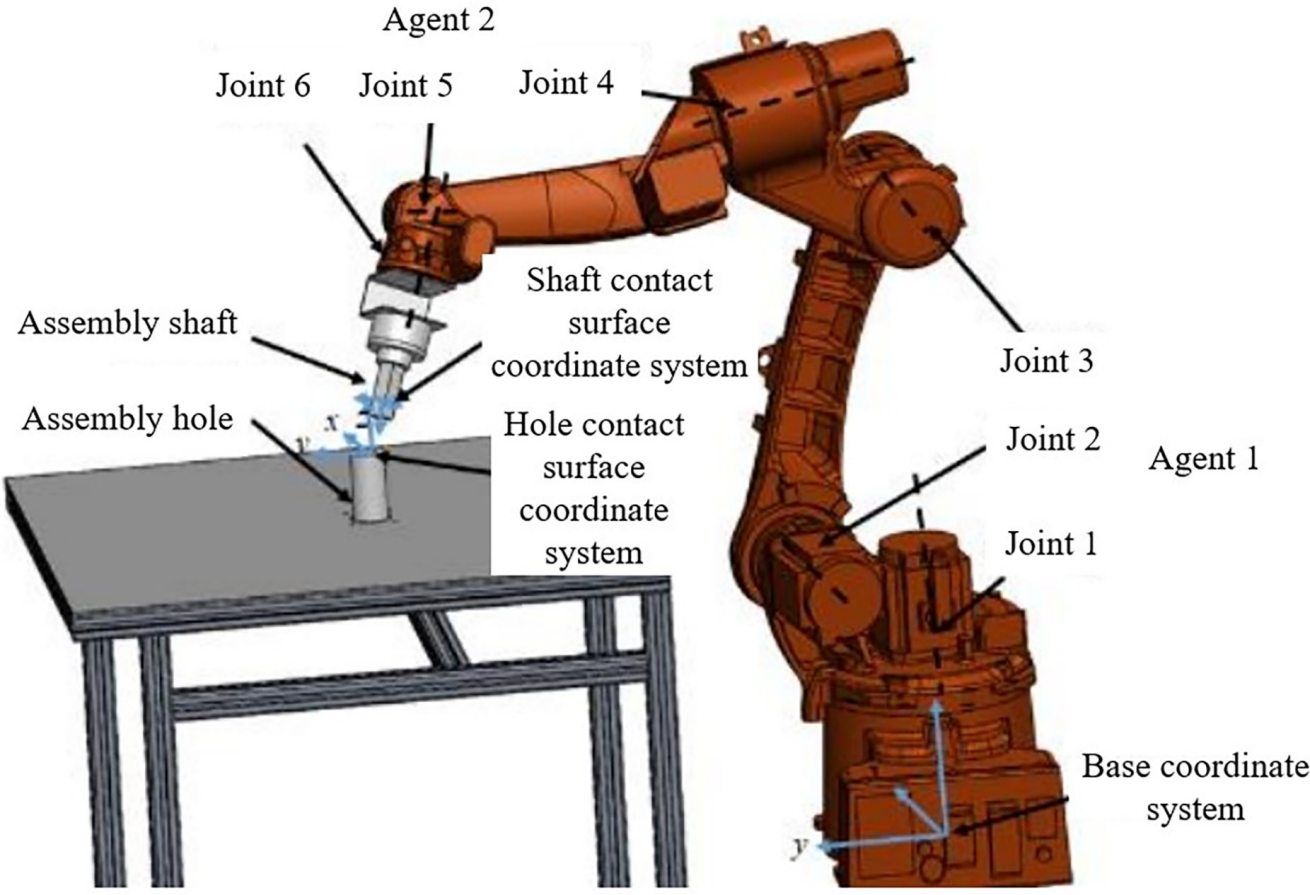
Schematic diagram of multi-agent axis assembly.

In the context of a multi-agent system that separates position and orientation control, the first three joints of the robotic arm are defined as agent 1, and the last three joints are defined as agent 2. The schematic diagram of multi-agent axis assembly is illustrated in **Fig 3**.

### 3.3 Decoupled multi-agent deterministic deep deterministic policy gradient algorithm

The MDDPG algorithm has been successfully applied in areas such as drones and multi-robot collaboration. However, in the context of multi-agent control of the serial robotic arms shown in **Fig 3**, strong coupling between these two agents leads to significant mutual influence during action execution, exacerbating competitive relationships among agents in cooperative tasks and hindering stable learning of appropriate joint strategies.

To address this issue, DMDDPG is proposed to reduce coupling between agents. DMDDPG is an enhanced version of MDDPG, utilizing a centralized training and distributed execution framework. In contrast to MDDPG, it incorporates not only a global Critic network for centralized training but also a local Critic network designed for each agent to evaluate only its own local states and actions. This design allows each agent to focus more on its own behavior, thereby enhancing the algorithm’s efficiency and stability. Each agent’s policy update considers not only the global optimal value function but also whether its own local value function is updating in the optimal direction. This decoupling approach prevents suboptimal and unstable solutions, as well as the dominance of global optimal rewards in the strategy among the group, ensuring each agent rapidly and effectively learns the optimal policy.

The training of the local Critic network only requires the current agent’s observations and states to evaluate its actions, obtaining a local *Q* value with the loss function:

$$L(\varphi_{i})=E_{o_{i},a_{i}∼D}[{\left(Q_{i}^{\pi }(o_{i},a_{i})-y_{\varphi }\right)}^{2}]$$ (14)

$$y_{\varphi }=r_{i}+\gamma Q_{i}^{\pi^{\prime }}(o_{i}^{\prime },a_{i}^{\prime })|a_{i}^{\prime }=\pi_{i}^{\prime }(a_{i}|o_{i})$$ (15)

where *φ*_*i*_ represents the parameters of the local Critic network for the *i*_th_ agent.

After introducing the local Critic network, updates to each agent’s Actor policy network require participation from both the global Critic network and the local Critic network to simultaneously explore global optimal solutions while exploring local optimal solutions. The policy gradient computation for the *i*_th_ agent then becomes:

$$\begin{array}{c} \nabla_{\theta_{i}}J(\theta_{i})=\underset{\mathrm{Global}\;\mathrm{value}\;\mathrm{function}}{\underset{︸}{E_{S,A∼D}[\nabla_{\theta_{i}}\log\pi_{i}(a_{i}|o_{i})Q_{i}^{H}(S,a_{1},\cdots a_{N})]}}\\ +\underset{\mathrm{Local}\;\mathrm{value}\;\mathrm{function}}{\underset{︸}{E_{o_{i},a_{i}∼D}[\nabla_{\theta_{i}}\theta_{i}(a_{i}|o_{i})\nabla_{a_{i}}Q_{i}^{\pi }(o_{i},a_{i})]}} \end{array}$$ (16)

The pseudo-code for the DMDDPG algorithm is shown in **Table 2**.

**Table 2:** Pseudo-code of DMDDPG algorithm.

Pseudo-code of DMDDPG Algorithm	
1	Initialize parameters of the global Critic main network $Q_{\theta }^{H}$.
2	Initialize parameters of the global Critic target network $Q_{\theta^{\prime }}^{H}$ and copy parameters $Q_{\theta }^{H}$.
3	Initialize Actor main network parameters $Q_{\theta_{i}}^{\pi }$ and Critic main network parameters $Q_{\varphi_{i}}^{\pi }$ for each agent.
4	Initialize Actor target network parameters $Q_{\theta_{i}^{\prime }}^{H}$ and Critic target network parameters $Q_{\varphi_{i}^{\prime }}^{\pi }$ for each agent, copy $Q_{\theta_{i}}^{\pi }$ and $Q_{\varphi_{i}}^{\pi }$ parameters.
5	**For** episode = 1 to MaxEpisode **do**
6	Initialize random process N, initialize environment state *S*.
7	**For** t = 1 to **do**
8	For each agent *i*, select action $a_{i}^{t}=\pi (o_{i}^{t}|\theta_{i}^{\pi })+N$.
9	Execute action $A^{t}=[a_{1}^{t},a_{2}^{t}]$.
10	Obtain global reward $R_{g}^{t}$, local reward $r_{i}^{t}$, and next state *S*^*t*+1^
11	Store sample $\left(S^{t},A^{t},R_{g}^{t},r_{i}^{t},S^{t+1}\right)$ into experience pool.
12	End for
13	Update global Critic network
14	Randomly sample *M* samples $\left(S^{t},A^{t},R_{g}^{t},S^{t+1}\right)$ from experience pool to form batch samples.
15	Calculate *y* from $y_{i}=r_{i}+\gamma Q^{\pi }(s_{t+1},a_{t+1})$.
16	Compute loss $\frac{1}{M}\sum_{i}{\left(y-Q_{\theta }^{H}(S^{t},A^{t})\right)}^{2}$ and update EvalCritic network $Q_{\theta }^{H}$ parameters.
17	Update target Critic network parameters $Q_{\theta^{\prime }}^{H}$ in a soft update manner.
18	Update robotic arm agent Actor network and local Critic network.
19	**For** agent = 1 to N **do**
20	Randomly sample *K* samples $\left(o^{t},a_{i}^{t},r_{i}^{t},o^{t+1}\right)$ from experience pool to form batch samples.
21	Obtain *y*_*φ*_ from Eq (15).
22	Compute loss $\frac{1}{K}{\sum_{i}\left(y_{\varphi }-Q_{\theta }^{H}(o_{i}^{t},a_{i}^{t})\right)}^{2}$ and update EvalCritic network $Q_{\varphi }^{\pi }$ parameters.
23	Update EvalActor network $Q_{\theta_{i}}^{\pi }$, network parameters *θ*_*i*_ as follows: $\theta_{i}=\theta_{i}+\frac{1}{K}\sum_{j}\left(\nabla_{\theta^{\pi }}\pi_{i}(a_{i}|o_{i})Q_{i}^{H}(S,a_{1},\cdots a_{N})+\nabla_{\theta^{\pi }}\pi_{i}(a_{i}|o_{i})Q_{i}^{\pi }(o_{i},a_{i})\right)$
24	**End for**
25	Update target Actor network parameters $Q_{\theta_{i}^{\prime }}^{\pi }$ and target Critic network parameters $Q_{\varphi_{i}^{\prime }}^{\pi }$ in a soft update manner.
26	**End for**
27	**End for**

### 3.4 Multi-agent action-state space definition

The design of the state space is crucial in deep reinforcement learning algorithms, as it defines the environment states that agents need to observe, enabling the value function to objectively evaluate the quality of actions. In the analysis of the shaft-hole assembly task in Section 1, the assembly completion is primarily judged by comparing the pose states between the shaft and hole contact surfaces. The assembly hole is fixed on the work platform, and its accurate pose state in the world coordinate system is known. The center point pose of the hole contact surface is defined as $F_{G}=[\begin{array}{cc} R_{G} & P_{G}\\ 0 & 1 \end{array}]$, and the pose information of the shaft’s contact surface is obtained from the forward kinematics of the robotic arm: $F_{M}(\theta )=[\begin{array}{cc} R_{M} & P_{M}\\ 0 & 1 \end{array}]$. To achieve the assembly goal, it is also necessary to ensure that the shaft is inserted to a specified depth *h*.

In the designed multi-agent algorithm, the joint state space *S* is defined as:

$$S=(o_{1},o_{2})=(F_{G},F_{M}(\theta ),h)$$ (17)

$$\left\{ \begin{array}{l} o_{1}=(P_{G},P_{M},h)\\ o_{2}=(R_{G},R_{M}) \end{array}\right.$$ (18)

where *o*_1_ represents the state space of Agent 1 controlling the end position state, where this agent also controls the depth of shaft-hole insertion. *o*_2_ represents the state space of Agent 2 controlling the end orientation state, focusing solely on posture matching during the assembly process.

Through the DMDDPG algorithm, the robotic arm autonomously learns to output the motion angles of each joint when faced with different state spaces, enabling the assembly shaft to smoothly achieve the assembly goal. Thus, the action spaces of the two agents are defined respectively as the rotation angles of joints 1–3 and joints 4–6. The joint action space *A* is defined as:

$$A=(a_{1},a_{2})$$ (19)

$$\left\{ \begin{array}{l} a_{1}=(\theta_{1},\theta_{2},\theta_{3})\\ a_{2}=(\theta_{4},\theta_{5},\theta_{6}) \end{array}\right.$$ (20)

### 3.5 Multi-agent reward function design

The shaft-hole assembly controlled by the robotic arm is a complex operation task in a continuous action space. The reward obtained to achieve the task goal is referred to as the main line reward. However, relying solely on the main line reward in high-difficulty exploration tasks can make it difficult for the algorithm to converge or result in slow convergence, a situation known as sparse reward problem. To overcome such issues, additional reward components need to be introduced, making the reward function dense. This helps guide the agents to explore the environment more efficiently, thereby accelerating the convergence speed and enhancing the performance of deep reinforcement learning algorithms. These types of reward functions are known as auxiliary rewards. Based on the different characteristics of the three stages—hole searching, alignment, and insertion—during the shaft-hole assembly process, different stage-specific reward functions are designed, mainly divided into sub-goal reward functions and shaping reward functions.

#### 3.5.1 Main line reward function

The main line reward is designed to set task objectives for reinforcement learning. In the execution of the shaft-hole assembly task, the reward is obtained only when (1) the shaft-hole posture matches completely, (2) the axis of the assembly shaft is aligned with the centerline of the hole, and (3) the shaft insertion depth reaches the specified depth, thus meeting the requirements for successful assembly.

**Fig 4:**
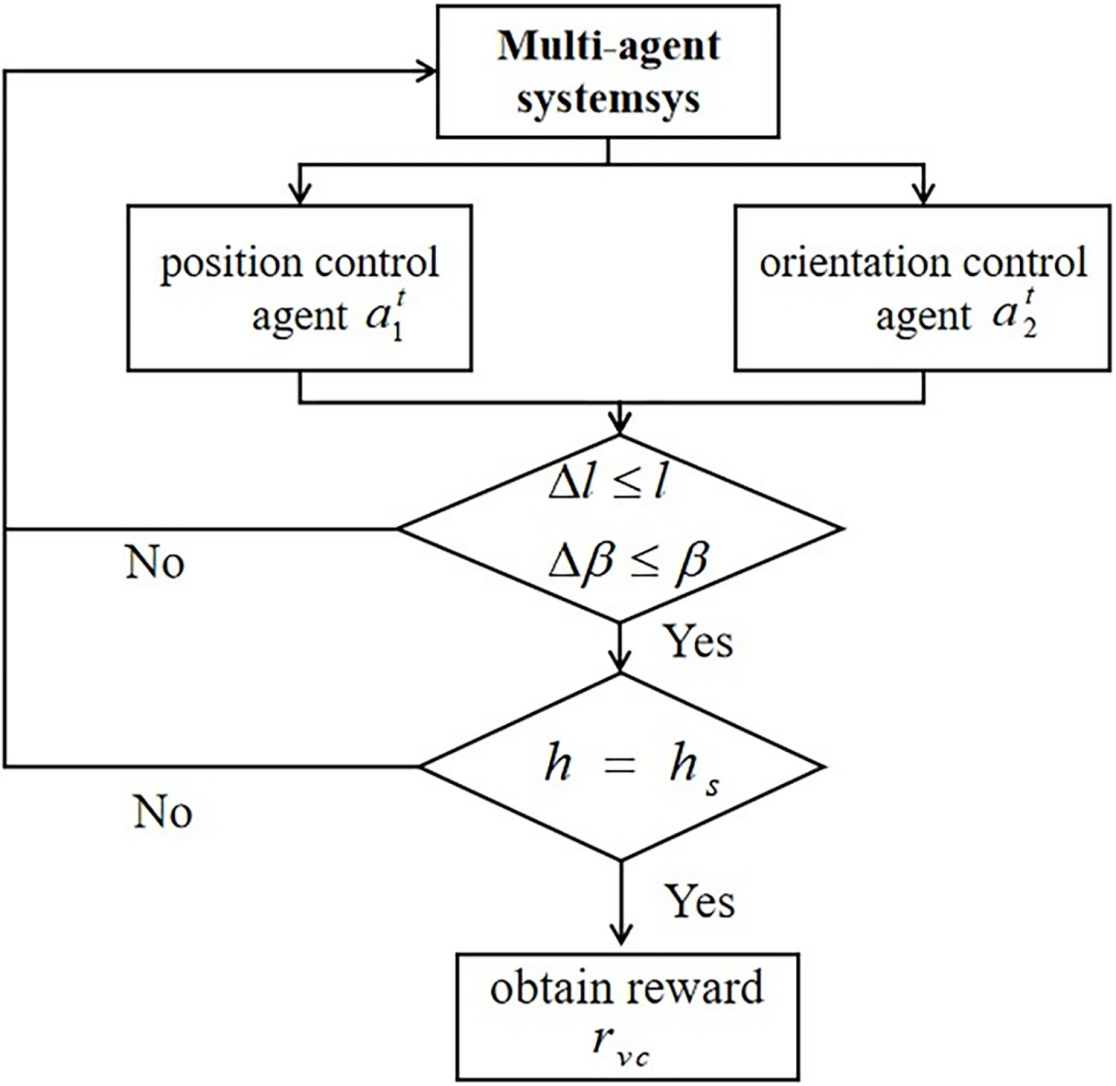
Assembly task flow.

At time *t*, each agent performs joint action $A^{t}=[a_{1}^{t},a_{2}^{t}]$, calculates the distance *S*^*t*+1^ between the shaft contact surface and the hole centerline, and the posture deviation Δ*l* of the shaft-hole assembly in state Δ*β*. If the specified assembly accuracy requirements are not met, the next iteration loop is carried out until the insertion requirements are fulfilled. During the shaft-hole insertion stage, the distance Δ*l* and posture deviation Δ*β* must remain within the error range (*l*,*β*). The task completion reward *r*_*vc*_ is obtained when the insertion depth *h*_*s*_ reaches the specified depth *h*, indicating successful assembly. The assembly task flowchart is shown in **Fig 4**.

The main line task reward can be defined as:

$$R_{g}=\{\begin{array}{l} r_{vc}(\Delta l\leq l,\Delta \beta \leq \beta ,h=h_{s})\\ \begin{array}{cc} 0 & otherwise \end{array} \end{array}$$ (21)

where *R*_*g*_ reflects the reward feedback only after successful assembly, serving as the signal to end each training round. If *R*_*g*_ is 0, the agent continues with the next action based on the error magnitude *r*_*vc*_ = 100 during training.

#### 3.5.2 Sub-goal reward functions

**Fig 5:**
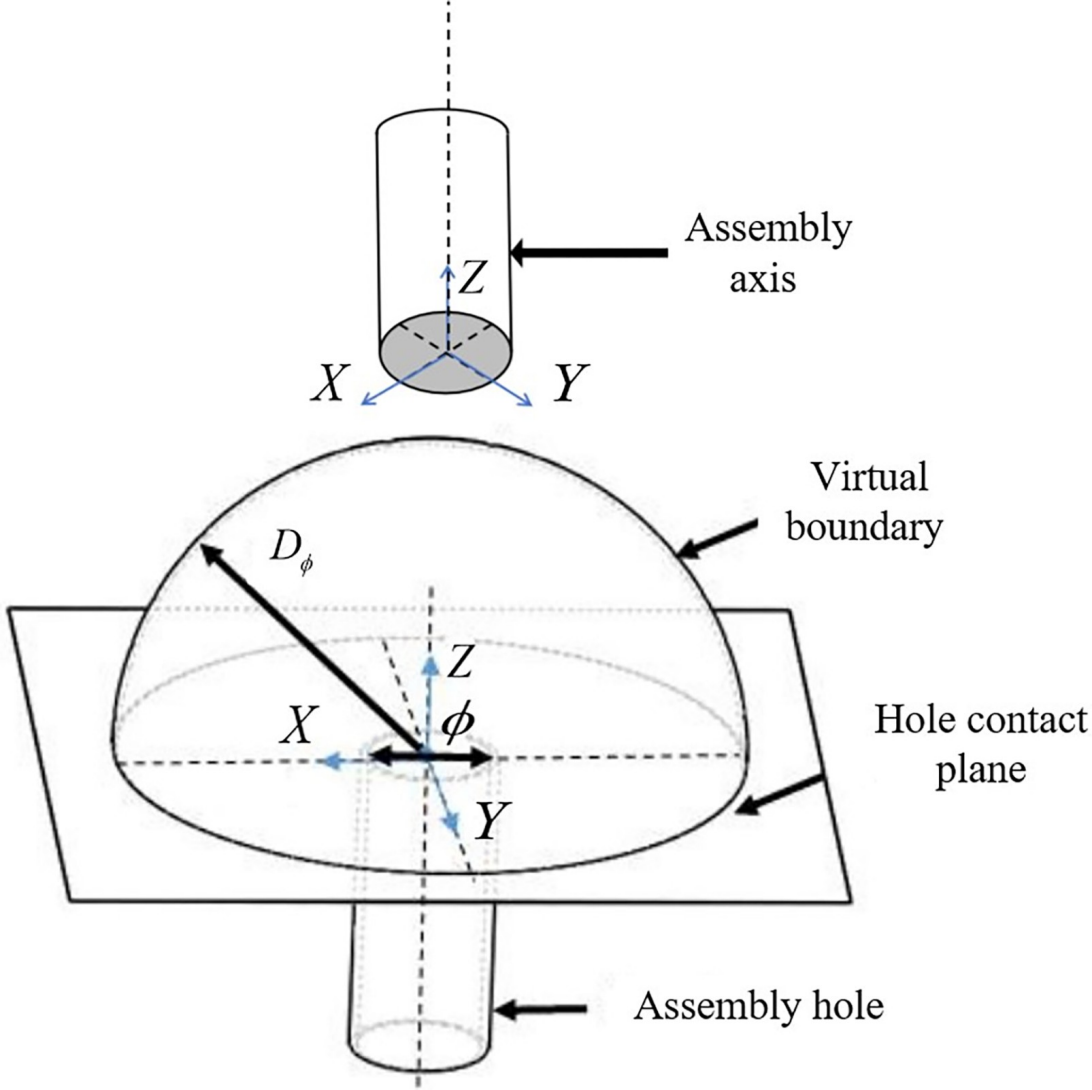
Shaft hole assembly virtual space.

Setting sub-goal rewards and other auxiliary rewards is primarily aimed at guiding the exploration process, narrowing down the ineffective exploration range of agents in the environment, and improving efficiency. In the exploration and completion of the main task, this process is further broken down into sub-goals, allowing the agents to first learn to accomplish these sub-goals. This approach increases the probability of subsequently exploring the main task, and finally, under the guidance of both main-line rewards and auxiliary rewards, completes the assembly task. To enhance assembly efficiency, penalty functions are introduced to ensure swift and safe achievement of each objective. Three sub-goal reward functions are primarily designed as follows:

1. Collision penalty function: Precision is crucial in the robotic assembly of shafts and holes, where collisions are strictly prohibited in industrial production and should be avoided during training. To enforce this principle, sensors measuring force/torque are installed between the robotic arm and the assembly shaft to detect real-time contact forces *F*. During assembly, forces or torques slowly change due to state modifications, but collisions cause sudden spikes. By setting a threshold *F*_lim_ for these spikes, collisions are detected when contact forces exceed this threshold, terminating the current exploration round and applying penalties. The penalty function *RF* is defined as shown in Eq (22), where feedback values *r*_*fl*_ = −100 are set during training.
$$R_{F}=\{\begin{array}{cc} r_{fl} & \left(F\geq F_{\lim}\right)\\ 0 & otherwise \end{array}$$ (22)
2. Angle penalty function: Continuous reward-penalty effects integrated into the exploration process typically outperform one-time rewards. Long-term accumulated rewards easily surpass sparse rewards, providing stronger guidance for algorithms. This approach offers immediate feedback directly correlated with current action status, benefiting neural networks in better feature extraction during the intermediate learning process. To improve exploration efficiency and prevent local oscillations that hinder convergence, the angle penalty function penalizes the rotational angles of each joint as quantitative targets. The angle penalty function is defined as *R*_*θ*_:
$$R_{\theta }=\ln(\sum_{i=0}^{6}|\theta_{i}|+1)$$ (23)
  where each joint’s rotational angle *θ*_*i*_ within one step is measured in radians. To avoid initial high penalties that hinder convergence, the logarithm of the sum of these six rotational angles within each step is used as the penalty value. Considering joint movement constraints and safety stability, the maximum allowable joint rotation angle is 3°, corresponding to *θ*_*i*_∈[0,0.052] and *R*_*θ*_∈[0,0.273].
3. Agent assembly virtual space:
  The task space for assembly is a relatively small region within the robotic arm’s larger operational workspace. Allowing the robotic arm to explore the entire workspace would be inefficient and result in a substantial amount of meaningless learning. Additionally, incorporating collision and angular penalties can lead the robotic arm into incorrect learning states. To better guide the completion of assembly tasks and enhance initial exploration efficiency, a spherical virtual space is defined with the center of the assembly hole’s contact surface as the origin, as illustrated in **Fig 5**. In the next section, reward functions for each agent will be defined based on their movement characteristics. This involves a continuous accumulation of rewards, encouraging agents to move closer to the task objectives to achieve higher rewards. To facilitate faster convergence to the assembly task area, rewards are accumulated only when the axis is within the defined virtual space. Outside this space, no rewards are given. Within the virtual space, positional and orientation deviations are compared, and positive rewards are provided to guide the agents more effectively towards successful assembly.

The virtual space is defined with a radius *VS*_*ϕ*_, determined by the basic dimensions of the assembly hole, and *VS*_*ϕ*_ = σ⋅*ϕ*. *ϕ* represents the diameter of the assembly hole, and *σ* is a scaling coefficient adjusted based on the dimensions of the shaft assembly and the robotic arm’s assembly environment. Through simulation experiments, it is found that when the coefficient is too large, initial rewards are high, but learning progress is slow, leading to lower final convergence rewards, which are suboptimal. Conversely, if the coefficient is too small, initial exploration times for objectives are extended, and timely adjustments to position and posture are hindered upon entry, making final convergence difficult. Through testing, a stable range for the virtual space radius coefficient is found to be *σ*∈(1.5,3). Within this range, the robotic arm can swiftly locate the target area and has ample time to adjust its position and posture.

#### 3.5.3 Shaping reward functions

Shaping functions are also auxiliary functions, different from sub-goal reward functions. Their main role is to add a type of potential energy function based on the agent’s state. They measure the gap between the agent’s state in the new environment after performing an action and the target state. The smaller the gap, the higher the corresponding reward, and vice versa. Reward functions need to be designed based on the tasks performed by each agent and the objectives they need to achieve. In shaft-hole assembly, localized reward functions are designed separately for agents controlling position and posture, termed as local reward functions.

**Fig 6:**
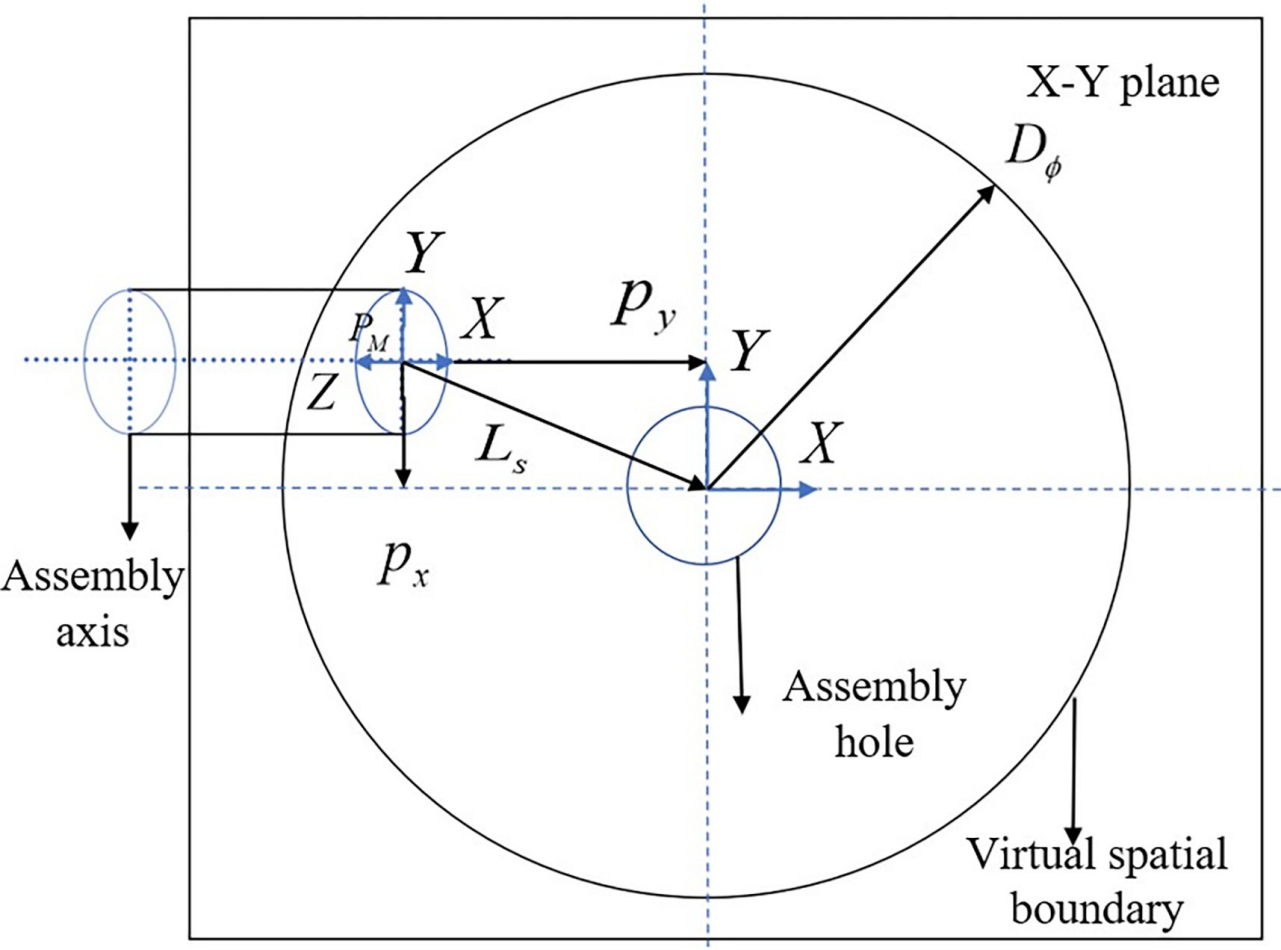
The projection distance between the center points of the shaft hole contact surface.

(1) Position-based reward function:
  In the assembly of square and circular shaft-holes, their requirements for position matching are the same. For ease of observation, let’s take the example of a circular shaft-hole to design a position-based reward function. For agents controlling only position, it is crucial to ensure that the center of the shaft contact surface is directly above the centerline of the hole. Therefore, the center point *P*_*M*_ of the shaft contact surface is projected onto the *X*-*Y* plane of the hole contact surface, and a reward function based on the distance *L*_*s*_ between the shaft and hole is designed, as illustrated in **Fig 6**. This distance is normalized based on the virtual space radius to ensure it does not change due to the size of the assembly hole and virtual space. The reward function *r*_*L*_ for horizontal distance is defined as:
$$r_{L}=1-\frac{L_{s}}{VS_{\phi }}=1-\frac{\sqrt{{\left(p_{M}^{x}-p_{G}^{x}\right)}^{2}+{\left(p_{M}^{y}-p_{G}^{y}\right)}^{2}}}{VS_{\phi }}$$ (24)
  where a smaller value of *L*_*s*_, indicating closer distance, results in a higher reward, and *r*_*L*_∈(0,1).

**Fig 7:**
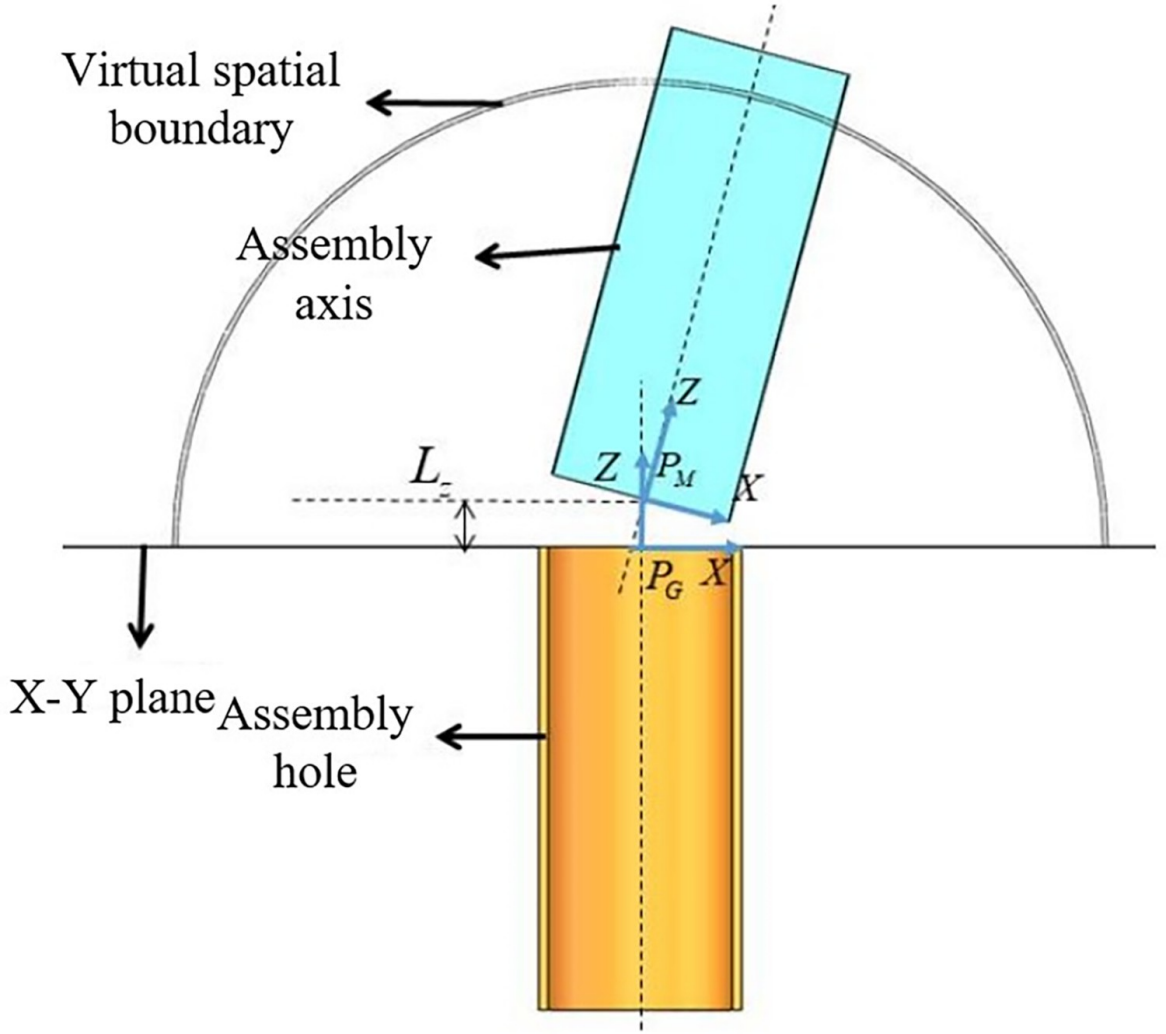
The vertical distance between the center points of the shaft hole contact surface.

Similarly, a reward function for vertical distance in the *Z*-axis direction is defined using the same method to prevent collisions between shaft-holes until posture matching is achieved by adding a weighting coefficient. The main calculation is the vertical distance *L*_*z*_ between the center points of the shaft-hole contact surfaces, as illustrated in **Fig 7**. The reward function for position matching phase is defined as *r*_*z*_:

$$r_{z}=\lambda (1-\frac{L_{z}}{VS_{\phi }})=\lambda (1-\frac{\left|p_{M}^{z}-P_{G}^{z}\right|}{VS_{\phi }})$$ (25)

where *λ*∈(0,1) is the weighting coefficient, and *r*_*z*_∈(0,*λ*). When *λ* is smaller, the reward value obtained when approaching in the *Z*-axis direction is smaller, and the guidance for the agent relative to movement in the *X*-*Y* plane is weaker, allowing the agent to prioritize horizontal position closer, providing more sufficient time for posture adjustment.

**Fig 8:**
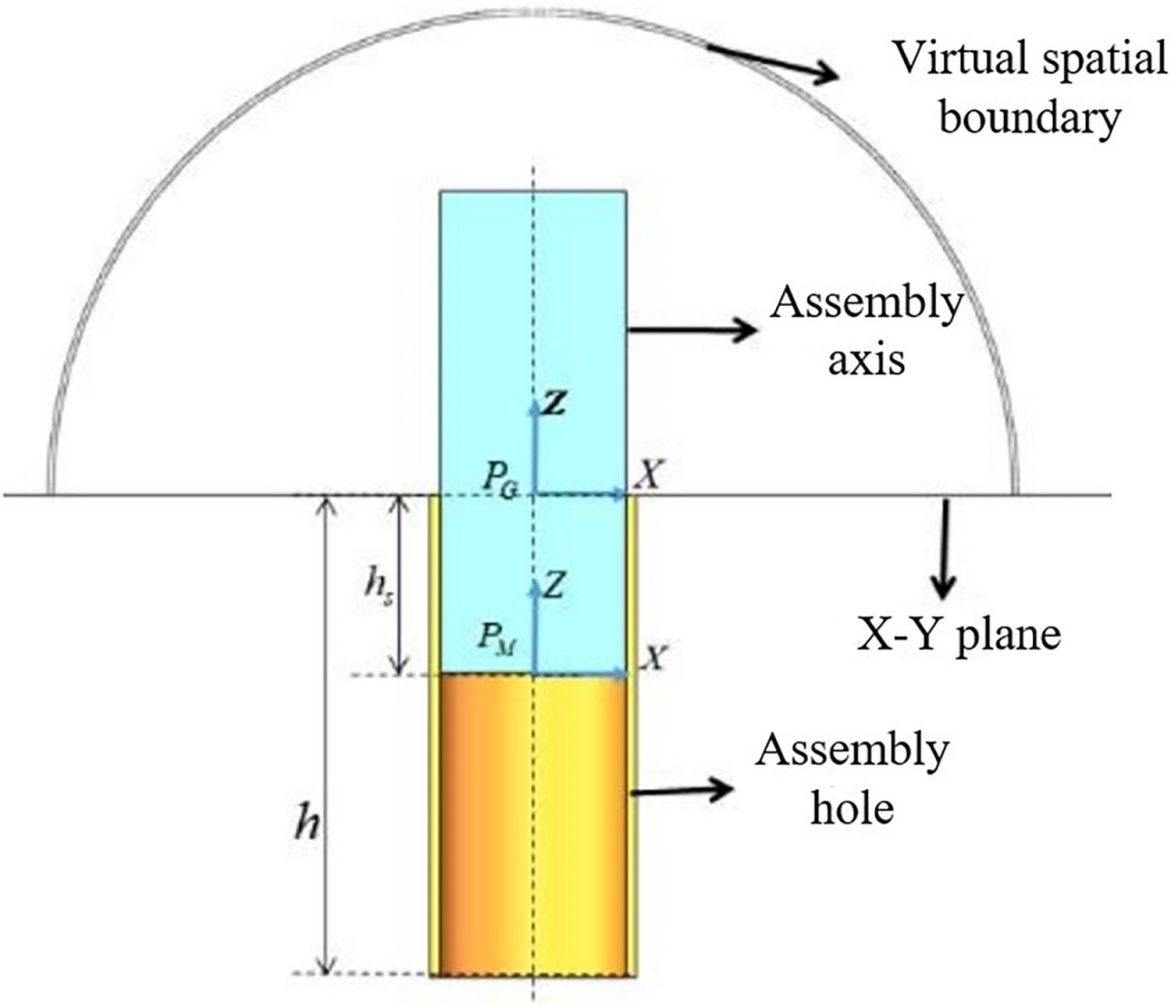
Shaft hole insertion.

After meeting the requirements for both position and posture, the insertion phase begins. During this process, the posture remains unchanged, and there is no movement in the *X*-*Y* axis direction. It is only necessary to observe whether the insertion depth *h*_*s*_ meets the assembly requirements, The insertion process is illustrated in **Fig 8**.

Insertion phase reward function is defined as:

$$r_{in}=e^{\left(1-|p_{M}^{z}-p_{M}^{z}|/h\right)}$$ (26)

where $|p_{M}^{z}-p_{G}^{z}|=h_{s}$, *r*_*in*_∈(l,*e*).

These three reward functions together constitute the position-based reward function. According to the shaft-hole assembly process, it can be divided into two stages: before and after insertion. The position reward function *R*_1_ is:

$$R_{1}=\{\begin{array}{l} r_{s}+r_{z},((p_{M}^{z}-p_{G}^{z})\geq 0)\\ r_{s}+r_{in},((p_{M}^{z}-p_{G}^{z})<0) \end{array}$$ (27)

where *r*_*s*_ and *r*_*z*_ will accumulate reward values in the virtual space after each step; *r*_*in*_ will accumulate rewards starting from the position and posture reaching requirements to execute the insertion phase, and *r*_*z*_ will no longer calculate reward values in this state. The range of values for *R*_1_ in these two stages is:

$$R_{1}\in \{\begin{array}{l} \left(0,1+\lambda )if((p_{M}^{z}-p_{G}^{z})\geq 0\right)\\ \left(1,e+1)if((p_{M}^{z}-p_{G}^{z})<0\right) \end{array}\}$$ (28)

(2) Pose-based reward function:

The assembly of square holes requires three directional deviation quantities to meet task requirements. Using a rotation matrix to represent the deviation between poses and the correction quantity will significantly increase the algorithm calculation volume. Therefore, quaternions are used to describe poses. Through Eq (11), quaternions can be converted into rotation matrices. Quaternions use only four elements to represent any pose vector in space, and the maximum angle between two vectors in opposite directions is *R*_2_. Thus, the pose reward function *π* is defined by the angle between quaternions:

$$R_{2}=\pi -\cos^{-1}(\left|q_{M}\cdot q_{G}\right|)$$ (29)

where *q*_*M*_ = (*ω*_*m*_,*a*_*m*_,*b*_*m*_,*c*_*m*_) is the pose of the shaft contact surface, *q*_*G*_ = = (*ω*_*g*_,*a*_*g*_,*b*_*g*_,*c*_*g*_) is the pose of the hole contact surface; *R*_2_∈(0,*π*), accumulating reward values begin after entering the virtual space until assembly completion.

In summary, the position reward function for agents controlling position is *R*_1_, and the pose reward function for agents controlling posture is *R*_2_. Their respective reward values range from different weight sizes. Posture adjustment is the most critical part, requiring agents to quickly and accurately adjust insertion posture and maintain it to achieve higher rewards. During the insertion along the hole centerline, posture adjustment and position closeness are encouraged with lower reward weight, allowing sufficient space and time. After contact, position reward weight is increased, encouraging agents to complete assembly tasks faster and better. Combined with main task rewards *R*_*g*_, collision penalty function *R*_*F*_, and angle penalty function *R*_*θ*_, the overall reward function for shaft-hole assembly based on deep reinforcement learning is:

$${\mathrm{Return}}^{t}=R_{g}+R_{F}-\sum_{t=0}^{T}R_{\theta }^{t}+\sum_{t=0}^{T}\gamma^{t}(R_{1}^{t}+R_{2}^{t})$$ (30)

where *Return*^*t*^ represents the total reward value obtained by the agent, and *γ* is the accumulated reward decay coefficient.

## 4. Axle hole assembly simulation experiment

### 4.1 Building the robotic arm simulation environment

#### 4.1.1 ROS system introduction

Robot Operating System (ROS) is an open-source system used for developing and controlling robots. It provides a structured way to build complex robot systems, where each functional module runs independently as nodes. These nodes are managed by a node manager (Master) that establishes connections between them using TCP/IP communication, enabling distributed network control. Nodes communicate by publishing (Talker) and subscribing (Listener) to messages, utilizing communication models such as topics and services to facilitate information exchange under different paradigms within ROS.

#### 4.1.2 Gazebo physics simulation platform

Gazebo is a 3D physics simulation platform that rapidly builds robot models. To enhance the realism of simulated robots, it interfaces with various 2D and 3D design software like CAD and SolidWorks. Detailed designs of robotic arm models in SolidWorks use the sw_urdf_exporter plugin to convert 3D models into URDF files, suitable for realistic modeling in simulation environments. Gazebo allows the addition of physical parameters such as mass, friction coefficients, inertia matrices, and collision properties to models, making the simulation environment more akin to real-world physics. Furthermore, Gazebo supports robot kinematics simulation, validating algorithms within constructed robots. To further match physical states realistically, Gazebo includes a comprehensive sensor model database featuring cameras, gyroscopes, scanners, and other commonly used sensors that can be directly added and invoked, supporting the creation of new sensors based on task requirements.

#### 4.1.3 Move It! motion planner

Move It! is an open-source software framework based on ROS for motion planning and control of robots. It offers a range of tools and libraries including motion planners, collision detectors, and motion controllers, facilitating rapid development of robot applications. The core node of the Move It! package is moving group, which, while not feature-rich on its own, integrates with other independent functional components to provide ROS action commands and services.

To achieve motion control of the robotic arm in an environment with known initial and target poses, the motion planner calculates appropriate motion trajectories guiding the arm to the target pose. Upon satisfying environmental constraints such as position, orientation, and velocity, the planner computes intermediate states.

#### 4.1.4 Building the robotic arm simulation model

**Fig 9:**
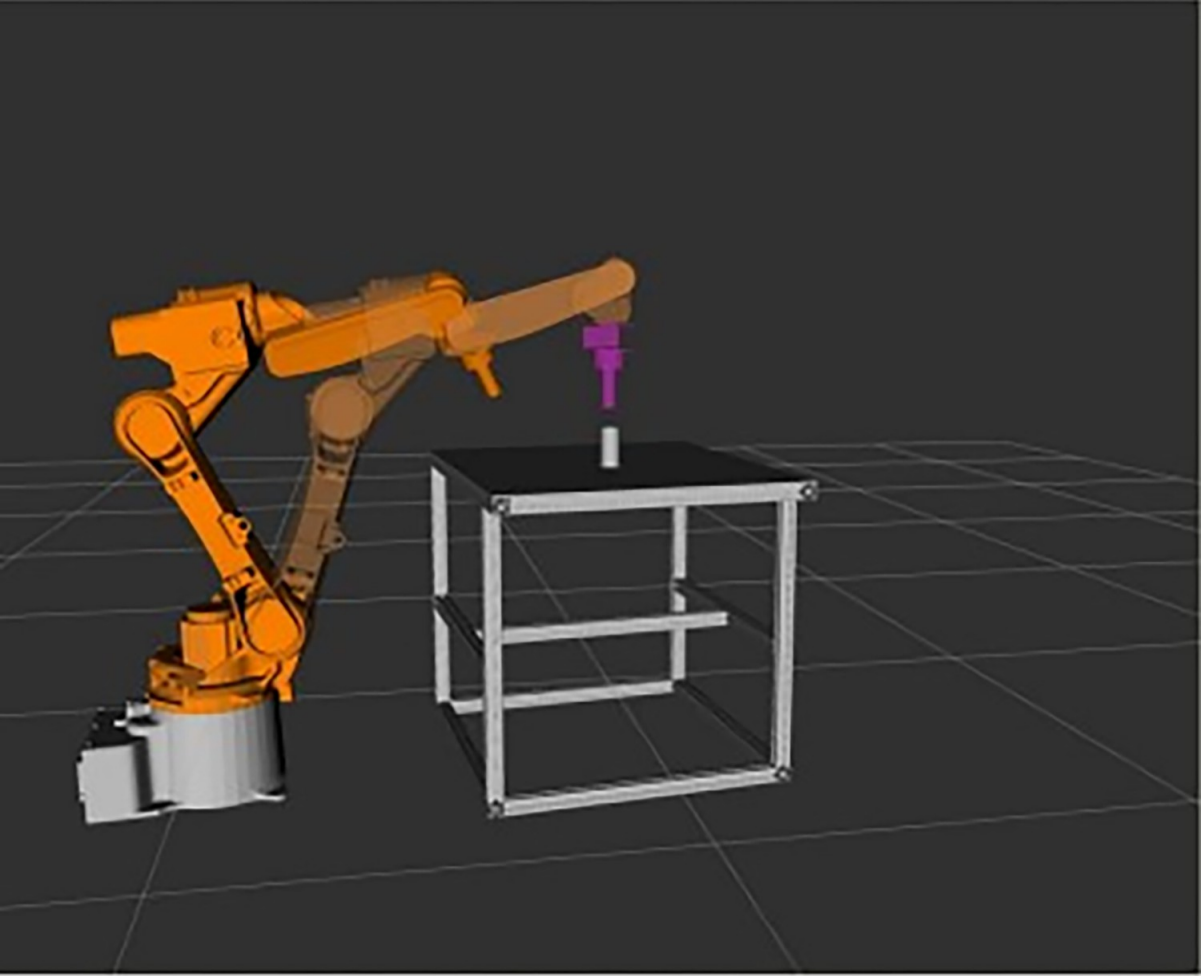
Robotic arm axle hole assembly model.

To build the robotic arm model in the Gazebo simulation environment, URDF files generated by 3D software are typically used for construction. These URDF files, written in XML, primarily describe the links and joints of the robotic arm. For simulation in Gazebo, it is necessary to add physical properties such as weight and inertia parameters (inertial tags) to each link, and collision detection 3D models (collision tags) to detect collisions. The robotic arm axle hole assembly model is displayed in the 3D visualization tool Rviz, as shown in **Fig 9**.

### 4.2 Assembly simulation training

#### 4.2.1 Network training parameters

In the DMDDPG algorithm, each agent has an Actor network and two Critic networks. The Actor network inputs the current observed state and outputs the value of each possible action in the current state, typically using the Tanh activation function in the output layer to scale the output values within the range of [−1,1]. The Critic network inputs the current state and action, and outputs the expected return of the agent, i.e., the Q value, given the state and action. To avoid training difficulties and overfitting, each network is designed as a three-layer fully connected neural network.

The training parameters of the DMDDPG algorithm for the axle hole assembly task are shown in **Table 3**.

**Table 3:** Network training hyperparameters.

Training parameters	Numerical value
Cumulative reward discount factor *γ*	0.99
Actor network learning rate *α*^*π*^	0.0001
Critic network learning rate *α*^*Q*^	0.001
Target network soft update rate *τ*	0.01
Simulation time step Δ*T*	0.1
Batchsize	64
Number of Episodes (MaxEpisode)	15000
Virtual space ratio *σ*	2

#### 4.2.2 Assembly experiment object

Section 3.1 analyzed the assembly processes for circular and square shaft-holes and explored the higher difficulty associated with square shaft-hole assemblies. A DE-MADDPG-based assembly method is proposed, which is also applicable to the simpler circular shaft-hole assembly. To validate the feasibility of the DE-MADDPG algorithm in a single robotic arm and its stability in handling tasks of varying difficulty, experiments are conducted using both circular and square shaft-holes as test objects.

**Fig 10:**
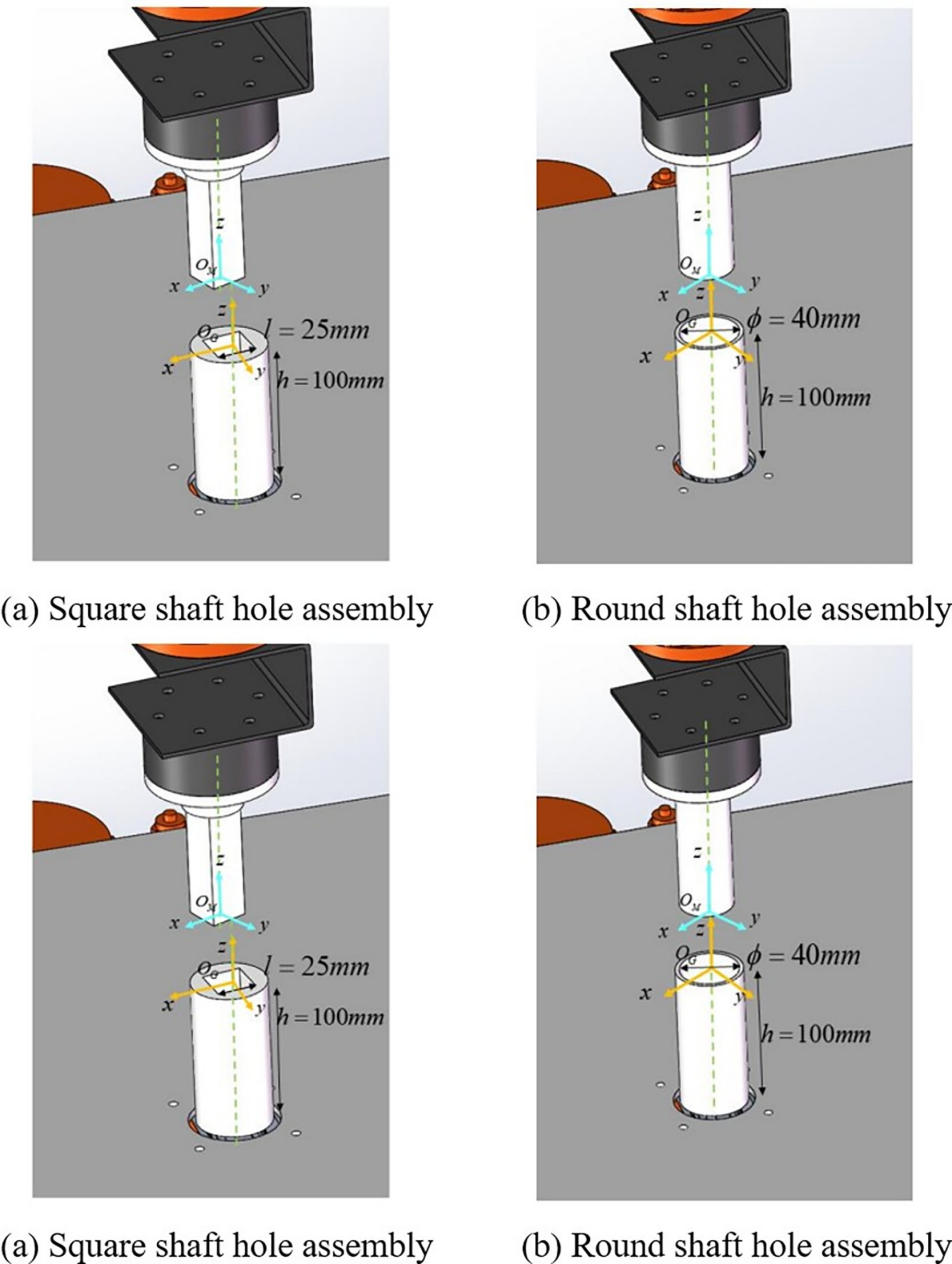
Assembling the subject. (a) Square shaft-hole assembly; (b) Circular shaft-hole assembly.

The specific structural parameters for the shaft-holes are as follows: For the square shaft-hole assembly, a square shaft-hole with a 25 mm edge length, an assembly clearance of 0.8 mm, and an insertion depth of 100 mm is used, as shown in **Fig 10A**. For the circular shaft-hole assembly, a base diameter of 40 mm, an assembly clearance of 0.8 mm, and an insertion depth of 100 mm are selected, as depicted in **Fig 10B**.

#### 4.2.3 Experimental results and analysis of DE-MADDPG shaft-hole assembly

**Fig 11:**
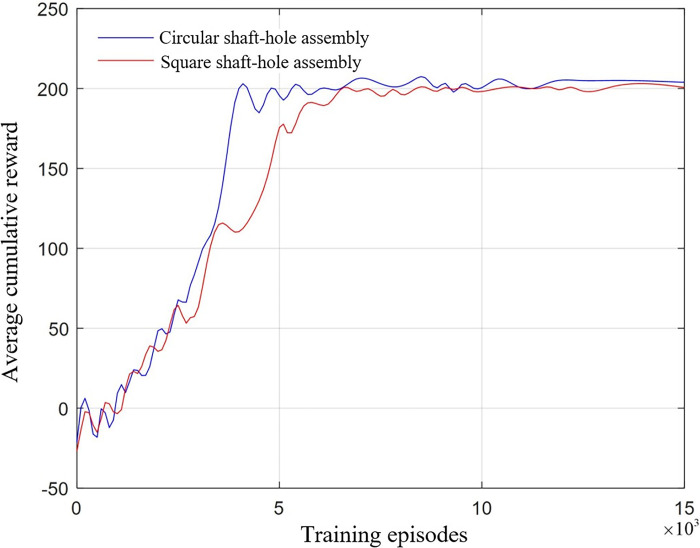
Reward curve of DE-MADDPG algorithm in circular shaft-hole and square shaft-hole assembly.

In the simulation control system established for the robotic arm assembly tasks, the DE-MADDPG algorithm is employed for the simulation training of both circular and square shaft-hole assemblies. After 15,000 iterations of learning, the cumulative average total rewards for the two assembly environments are shown in **Fig 11**.

The reward values for the algorithm converged to a stable state in both assembly environments, demonstrating the feasibility of decomposing a single robotic arm into two agents—one controlling the end-effector position and the other managing orientation—and coordinating their control using the DE-MADDPG algorithm.

As observed in the figure, during the initial exploration phase of approximately 3,000 episodes, the reward value curves for both assembly tasks are similar. During this phase, the agents primarily learned to quickly locate the hole through spatial exploration from the initial configuration. In the subsequent alignment and insertion phases, the circular shaft-hole assembly task, which requires less stringent orientation control, saw its reward values increase rapidly and stabilize around episode 4,500, with a final stable average reward of 202.52. In contrast, the square shaft-hole assembly task required multiple rounds of learning to achieve the desired pose matching after correctly locating the hole. Consequently, the reward values began to stabilize around episode 6,000, with a final average reward slightly lower than that for the circular shaft-hole assembly, at 199.46.

### 4.3 Comparative experiment analysis

#### 4.3.1 Comparative assembly experiment of DMDDPG and DDPG

DDPG, as a single-agent algorithm, is widely and maturely applied in axle hole assembly tasks, but most assembly objects primarily involve circular axle holes. The circular axle hole and square axle hole assembly tasks are executed using the same parameters as the DMDDPG algorithm to comparatively analyze the performance differences between the multi-agent algorithm and the single-agent algorithm in controlling the assembly task of a single robotic arm.

**Fig 12:**
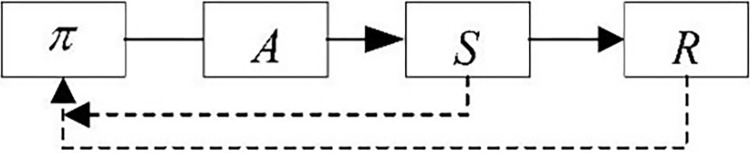
Simplified DDPG process.

DDPG, as a single-agent algorithm, evaluates action *A* using the overall environment *S* and feeds back the total reward value *R* to the agent to update the action policy. The algorithm process can be simplified as shown in **Fig 12**.

**Fig 13:**
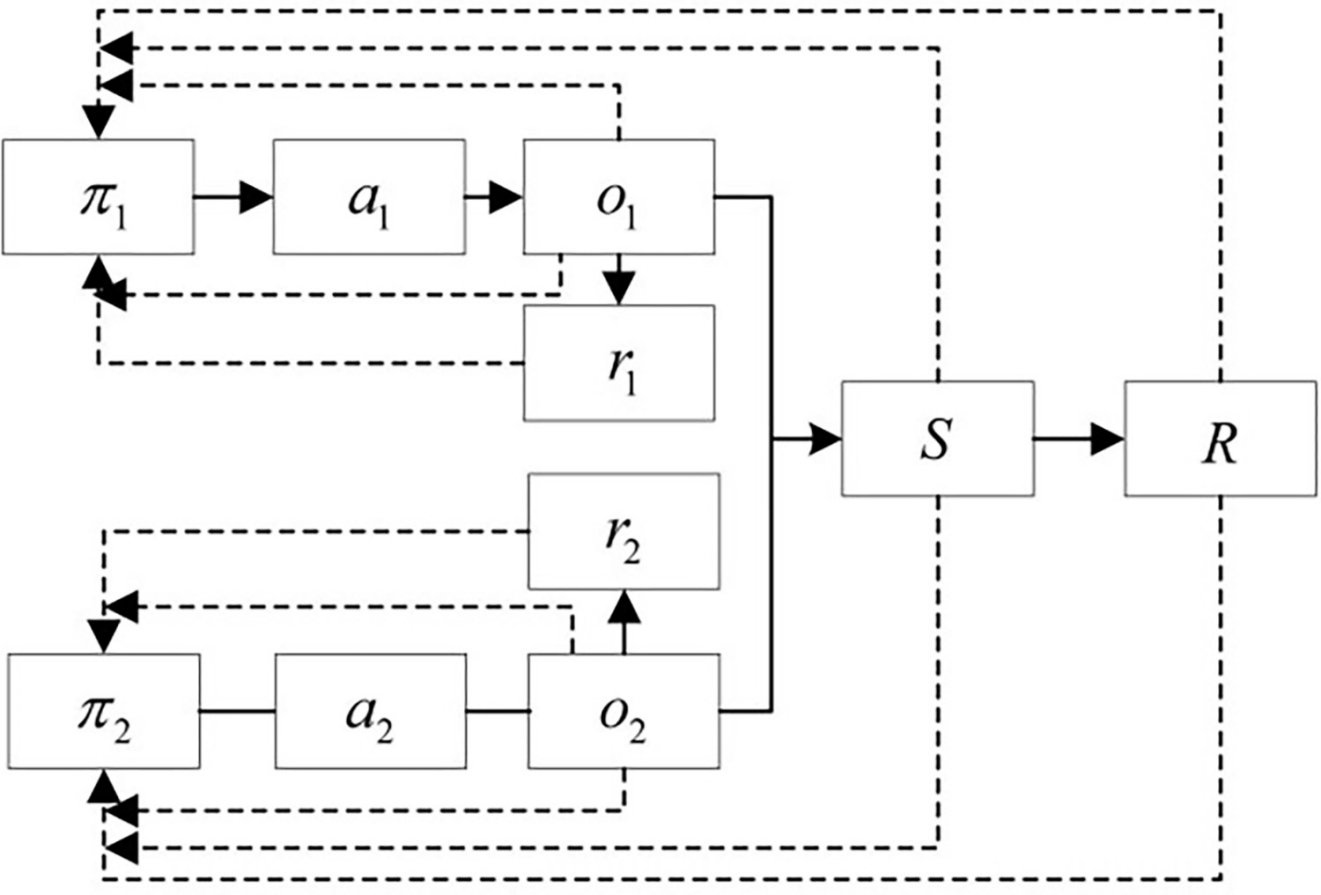
Simplified DMDDPG process flowchart.

To intuitively compare the differences with the DMDDPG algorithm, according to the DMDDPG algorithm shown in **Table 2**, it is simplified into the algorithm flowchart shown in **Fig 13**.

The total reward value *R* includes the local rewards *r*_1_ and *r*_2_ of the agents, i.e., local rewards. During training, multi-agents use not only the overall environment *S* and total reward value *R* but also the agent’s own observation environment and state *o*_*i*_, as well as local reward values to jointly guide the agent’s policy update. This is the biggest difference between the DMDDPG algorithm and the DDPG algorithm.

For consistency in evaluation standards, DDPG also learns and trains using the total reward function defined in Eq (30), with state space *S* = (*F*_*G*_,*F*_*M*_(*θ*),*h*) and action space *A* = (*θ*_1_,*θ*_2_,*θ*_3_,*θ*_4_,*θ*_5_,*θ*_6_).

**Fig 14:**
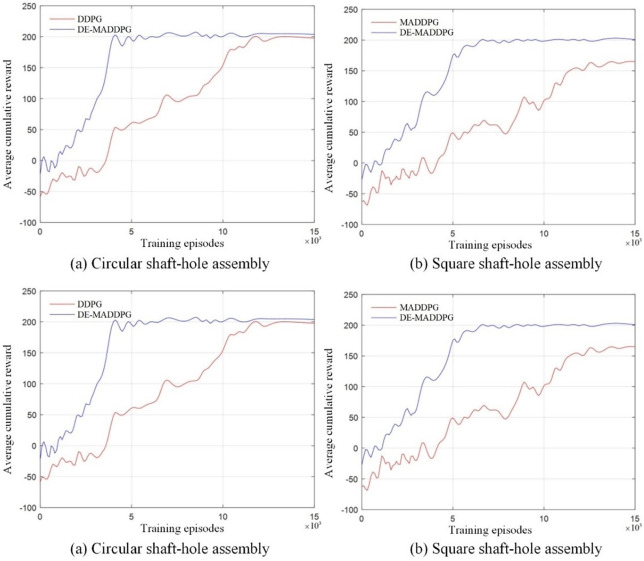
Cumulative average total reward value of DMDDPG and DDPG.

The DDPG algorithm, designed with the network parameters outlined in **Table 3**, is applied to the training of both circular and square shaft-hole assemblies. The cumulative average total rewards achieved with the DDPG algorithm are compared to those obtained with the DE-MADDPG algorithm, as illustrated in **Fig 14**.

The DDPG algorithm’s final training results show convergence to a stable state. However, during the initial exploration phase, it lacked accurate control over position and orientation, leading to blind operation in the space. Consequently, each iteration reward value is approximately 40 points lower than that of the DE-MADDPG algorithm and increased slowly during later stages of exploration.

In the circular shaft-hole assembly (**Fig 14A**), the DDPG algorithm began to stabilize around 12,000 episodes, requiring three times as many training cycles compared to DE-MADDPG. The final reward value converged to 198.43, showing minimal difference from DE-MADDPG, which indicates that both algorithms learned similar optimal assembly strategies. For the square shaft-hole assembly (**Fig 14B**), the DDPG algorithm’s reward values also show a convergence trend around 12,000 episodes, but continued to increase slightly until the end of the training period and had not fully stabilized. The final reward value is approximately 165, about 35 points lower than that of DE-MADDPG.

The comparative experiments highlight that the DE-MADDPG algorithm has a clear advantage in training efficiency. It maintains stable performance across tasks of varying complexity, demonstrating its superior effectiveness in assembly tasks compared to the DDPG algorithm.

#### 4.3.2 Comparative assembly experiment of DMDDPG and MDDPG

**Fig 15:**
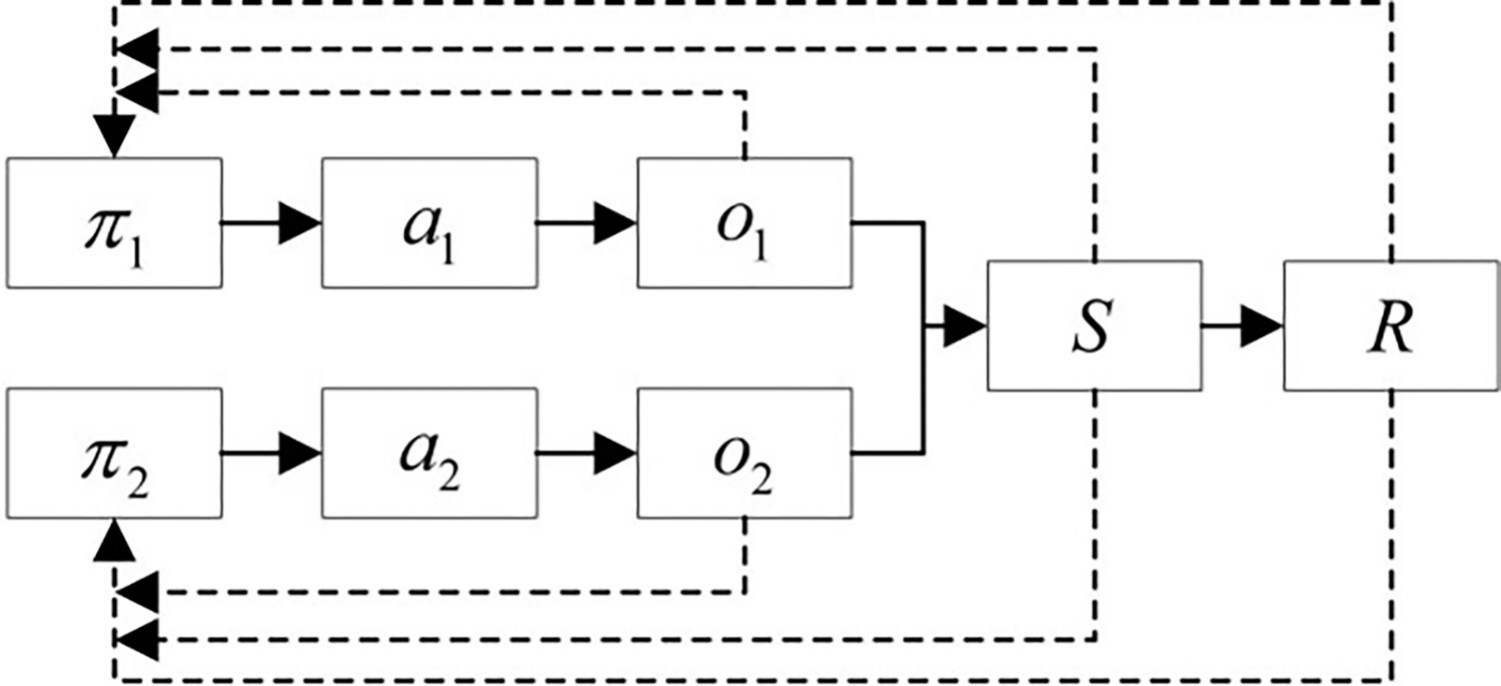
Simplified MDDPG process.

The DMDDPG algorithm is based on the MDDPG algorithm, designed to address the coupling between multiple agents by incorporating local evaluation functions for decoupling. By applying the MDDPG algorithm to the same assembly task, we can compare the performance changes brought by the addition of local evaluation functions. The training process of the MDDPG algorithm is simplified as shown in **Fig 15**.

**Fig 16:**
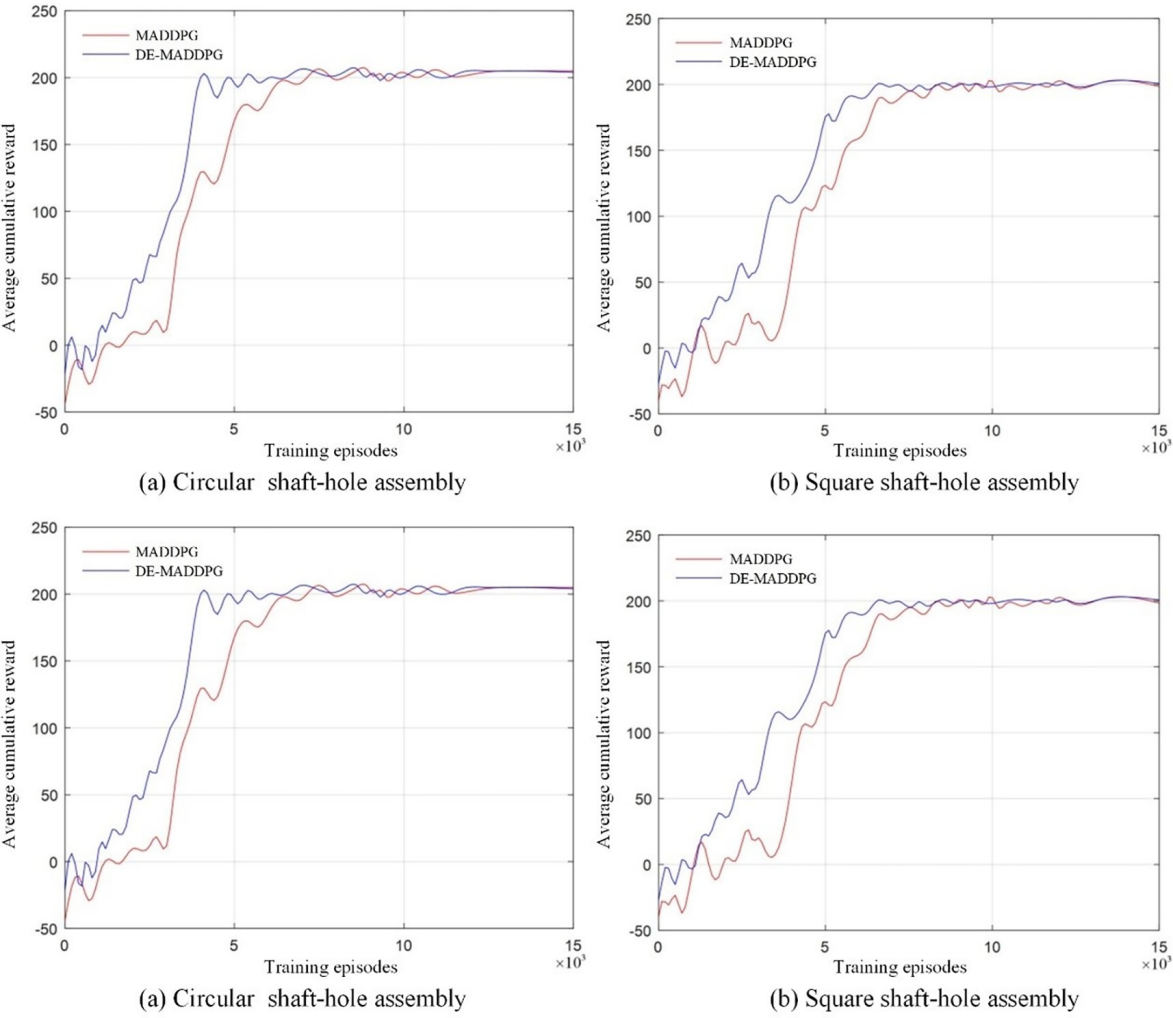
Cumulative average total reward value of DMDDPG and MDDPG.

Compared to the simplified DMDDPG process shown in **Fig 15**, the only difference is the absence of the local reward function evaluation. The action state space (*A*,*S*) and the reward (*R*) definitions are consistent with the DMDDPG algorithm. Using the same network parameters, the MDDPG algorithm is employed for the assembly training of circular shaft-hole and square shaft-hole. The comparison of the cumulative average total reward values is shown in **Fig 16**.

As shown in **Fig 16**, the final convergence states of the DE-MADDPG and MADDPG algorithms are nearly identical, which demonstrates the superiority of the multi-agent approach. However, MADDPG exhibited lower initial exploration rewards compared to DE-MADDPG, and reward value increases are not particularly significant before 3,000 episodes. This is due to the system’s inability to effectively allocate tasks to individual agents under the guidance of only global rewards, coupled with strong competitive interactions between agents. Consequently, the MADDPG algorithm required approximately 3,000 additional training episodes to reach a convergence state compared to DE-MADDPG. In the square shaft-hole assembly (**Fig 16B**), the presence of coupling led to considerable fluctuations in reward values once convergence is achieved, indicating system instability. The introduction of the decoupling module in the DE-MADDPG algorithm allows it to better handle environments with strong coupling between agents, enhancing training efficiency and improving system stability.

### 4.4 Assembly experiment validation

Three algorithms are used for the assembly training of the square axle hole, all of which achieved stable convergence. To verify the effectiveness of the algorithm strategies, 500 assembly tests for circular and square axle holes are conducted in the simulation environment using the DDPG, MDDPG, and DMDDPG algorithms. The test results are shown in **Table 4**.

**Table 4:** Comparison of assembly results of three algorithms.

Assembly algorithm	Circular shaft-hole assembly			Square shaft-hole assembly		
	Time per assembly(s)	Standard deviation	Success rat%	Time per assembly(s)	Standard deviation	Success rat%
DDPG	56.45	6.27	83.8	62.86	7.85	76.2
MADDPG	52.71	4.38	89.2	57.02	5.43	84.6
DE-MADDPG	51.55	3.86	92.4	54.58	4.12	87.8

The standard deviation in **Table 4** reflects the variability in execution time for assembly tasks, with a smaller value indicating greater stability in the algorithm’s performance across different environments. In the circular shaft-hole assembly, both multi-agent algorithms (DE-MADDPG and MADDPG) demonstrates improvements in total assembly time compared to the DDPG algorithm, with enhancements of 8.7% and 6.6%, respectively. Additionally, the standard deviation of the average single-assembly time is significantly reduced for the multi-agent algorithms, indicating greater stability in the assembly process. For the square shaft-hole assembly, the DE-MADDPG algorithm shows a notable improvement over the DDPG algorithm, reducing the single-assembly time by 8.28 seconds and increasing assembly efficiency by 13.2%. Although the standard deviation of the single-assembly time increased slightly compared to the circular shaft-hole assembly, it remained within acceptable limits. In both assembly environments, multi-agent algorithms consistently achieved assembly success rates above 80%. Notably, the DE-MADDPG algorithm achieves a success rate of 92.4% in the circular shaft-hole assembly.

## 5. Assembly test rig experiment

### 5.1 Setup of the test rig

#### 5.1.1 Components of the test rig

**Fig 17:**
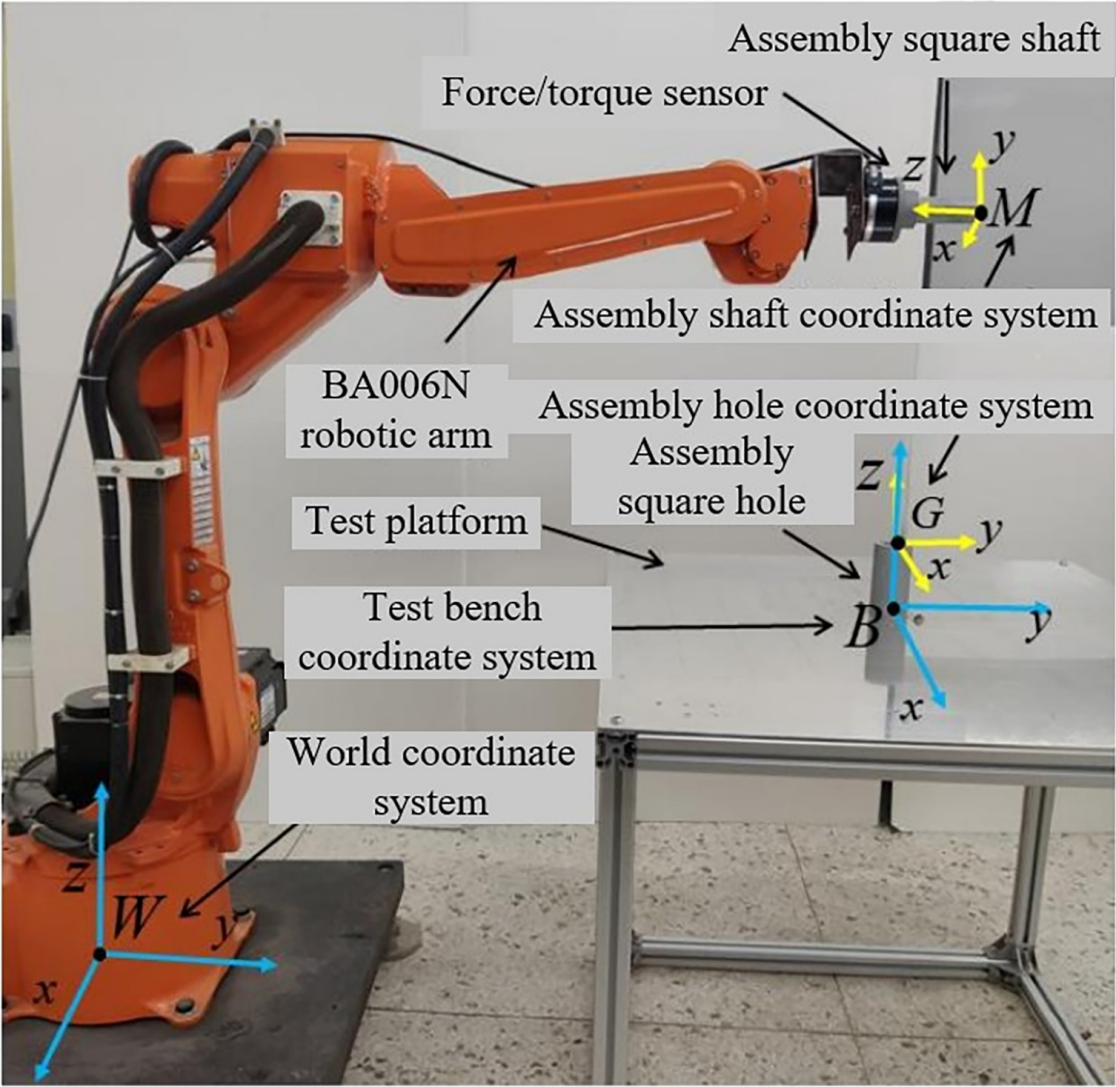
Test rig for square shaft-hole assembly.

For this experiment, a Kawasaki BA006N robotic arm equipped with a motion controller is selected as the primary control object. The origin of the world coordinate system was set at the base of the robotic arm. An assembly platform is constructed, with the platform’s center point’s coordinate parameters determined within the world coordinate system. The assembly object is a square shaft-hole with a side length of 25 mm, an assembly depth of 100 mm, and an assembly clearance of 0.8 mm, positioned at the center of the assembly platform. The test rig setup is shown in **Fig 17**.

The Kawasaki BA006N robotic arm has a repeatability precision of ±0.06 mm and a maximum payload of 6 kg, making it suitable for the assembly tasks. A force/torque sensor is installed between the assembly shaft and the robotic arm to detect collisions, with the collision threshold *F*_lim_ determined through contact experiments discussed in the next section. During each training session, the joint angles of the robotic arm’s initial posture are fixed at $\theta =[0^{\circ },0^{\circ },0^{\circ },0^{\circ },0^{\circ },0^{\circ }]$, with the initial configuration shown in **Fig 17**. A coordinate system *M* is established at the contact surface of the assembly shaft’s endpoint as depicted in **Fig 17**. The position and orientation information *F*_*M*_(*θ*) of the contact point center in the world coordinate system at the initial state is determined.


$$F_{M}(\theta )=\left[\begin{array}{cc} R_{M} & P_{M}\\ 0 & 1 \end{array}\right]=[\begin{array}{cccc} 0 & -1 & 0 & 0\\ 0 & 0 & -1 & 1.3\\ 1 & 0 & 0 & 1.19\\ 0 & 0 & 0 & 1 \end{array}]$$ (31)

The test rig is installed in a fixed location, and the position of the testing platform’s center point within the world coordinate system is determined through the calibration of the robotic arm, establishing the rig’s coordinate system B. The assembly hole is mounted at the center point, and a coordinate system G is established at the contact point center of the assembly hole based on the structural dimensions. The position and orientation information *F*_*G*_ of the contact point center of the assembly hole in the world coordinate system are then determined.


$$F_{G}=\left[\begin{array}{cc} R_{G} & P_{G}\\ 0 & 1 \end{array}\right]=[\begin{array}{cccc} 1 & 0 & 0 & 0\\ 0 & 1 & 0 & 1.2\\ 0 & 0 & 1 & 0.85\\ 0 & 0 & 0 & 1 \end{array}]$$ (32)

According to the analysis of orientation in Section 3.2, for ease of calculation, expressions *R*_*M*_ and *R*_*G*_ are converted into quaternion form, resulting in the orientations *q*_*M*_ and *q*_*G*_ or the shaft and the hole, respectively.


$$\left\{ \begin{array}{c} q_{M}=(0.5âˆ’0.50.5âˆ’0.5)\\ q_{G}=(1\;0\;0\;0) \end{array}\right.$$ (33)

#### 5.1.2 Sensor collision threshold design

A force/torque sensor, installed between the assembly shaft and the robotic arm, is employed to detect collisions between the shaft and the surrounding environment. The sensor’s maximum range and precision are detailed in **Table 5**.

**Table 5:** Force/Torque sensor parameters.

Force/torque	Range	Precision
*F*_*xyz*_(*N*)	2000	1
*M*_*xyz*_(*N*⋅*m*)	60	0.01

Before the experiment, the sensor is calibrated in a no-load state. Once the assembly shaft is attached, it introduces a certain load force and torque. During the movement of the robotic arm, changes in the endpoint shaft’s state will lead to variations in the force and torque. The motion control algorithm imposes restrictions on the allowable rotational angle within each step to minimize the influence of acceleration on force and torque during small-angle continuous rotations. Thus, the force and torque changes observed by the sensor are related solely to the shaft’s current state. During normal movement, the rate of change in force and torque over a unit of time should remain within a certain range. Exceeding this threshold indicates a sudden change in the shaft’s state, signaling a collision.

The collision threshold is crucial for the success of the shaft-hole assembly. If the threshold is set too low, even slight contact with the environment would trigger a collision penalty and terminate the training. This could prevent the agent from learning effective optimization strategies, making it difficult for the algorithm to converge. Conversely, if the threshold is set too high, the system might continue exploring even after contact, leading to irreversible damage to the assembly shaft-hole if feedback adjustments are not timely.

In practical scenarios, it is necessary to set an appropriate collision threshold through preliminary experiments. During the approach phase of the assembly process, the shaft might collide with the working environment, which is common in early exploration stages. After sufficient learning, these collisions can be effectively avoided. During the search and insertion phases, contact primarily occurs between the shaft and the hole, which persists until the algorithm stabilizes. Additionally, the complex conditions during the square shaft-hole assembly process need particular attention.

**Fig 18:**
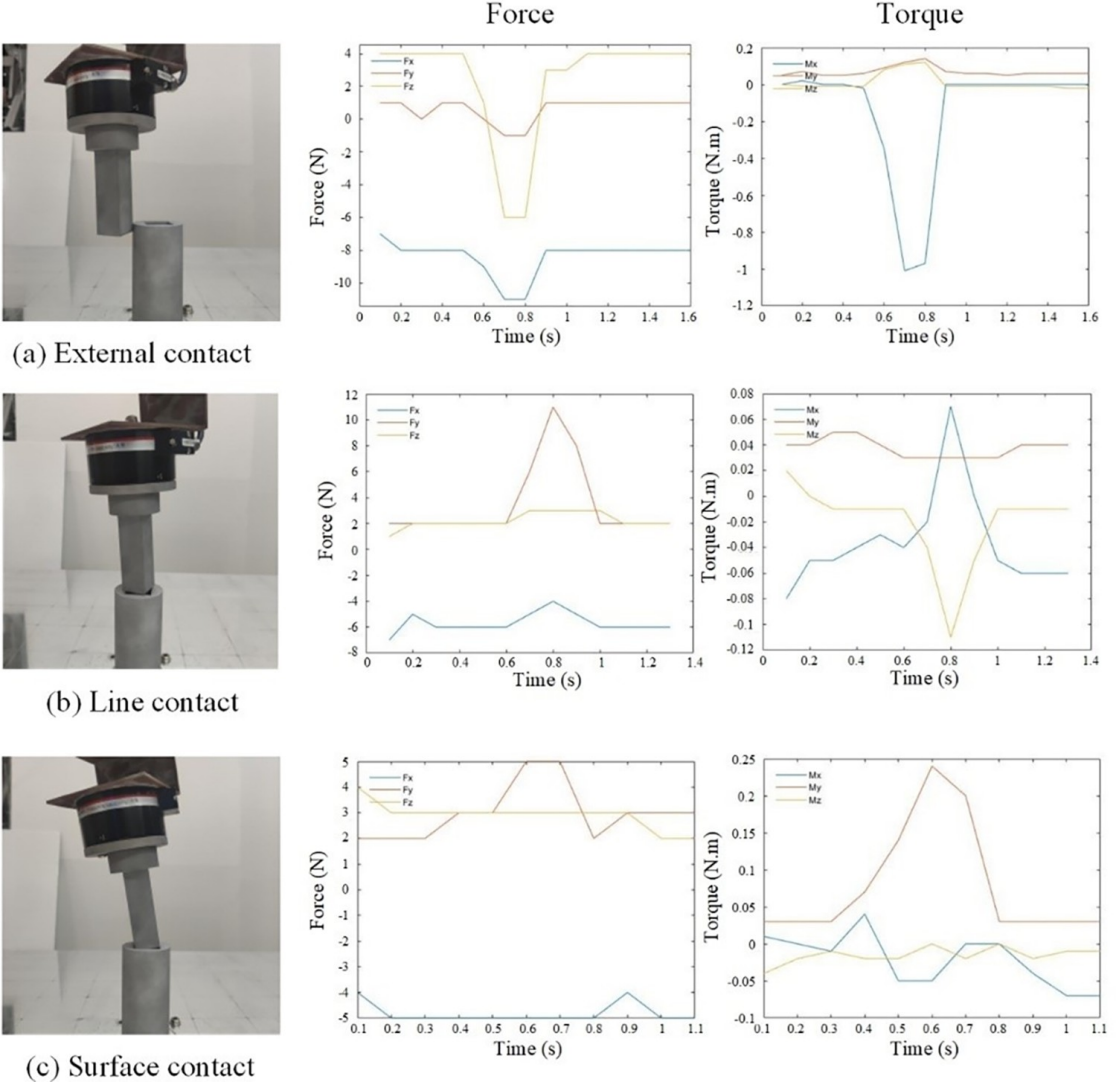
Force variations under different contact states.

To assess different force conditions, the square shaft-hole is tested in various contact states. The hole is placed in a fixed position without being secured, and significant tipping is considered as a collision state. Three experiments are conducted: (a) External wall contact: This simulates collisions between the shaft and the environment during the search phase. (b) Contact with the hole surface: This corresponds to the pose alignment phase. (c) Line contact with the hole edge: This occurs during the insertion phase, where collisions with the hole wall are likely. The corresponding force and torque variations for these contact states are shown in **Fig 18.**

In the outer contact state, both force and moment undergo significant changes, with the maximum variations being Δ*F*_*z*_ = 10*N* and Δ*M*_*x*_ = 1*N*⋅*m*. During surface contact, positional and angular deviations occur, with the *y*-axis force showing the largest variation at Δ*F*_*y*_ = 11*N*, and the moments along the *x* and *z* axes changing significantly, denoted as Δ*M*_*x*_ = Δ*M*_*z*_ = 0.11*N*⋅*m*. In the insertion phase, where line contact is observed, the main issue is angular deviation, with minimal force variation and a maximum moment change of Δ*M*_*y*_ = 0.23*N*⋅*m*.

Based on the above analysis, it is crucial to ensure that both force and moment remain within safe limits during the assembly process. To prevent system misjudgments due to changes in force and moment induced by the robotic arm’s motion, the collision condition *F*_lim_ is set as the maximum rate of change of contact force and moment per unit time step, resulting in conditions *F*_max_ = 50*N*/*s* and *M*_max_ = 0.5*N*⋅*m*/*s*. When the rates of change in force and moment in all three directions are within acceptable ranges, the action within the current time step is deemed safe and feasible.

### 5.2 Experimental results analysis

**Fig 19:**
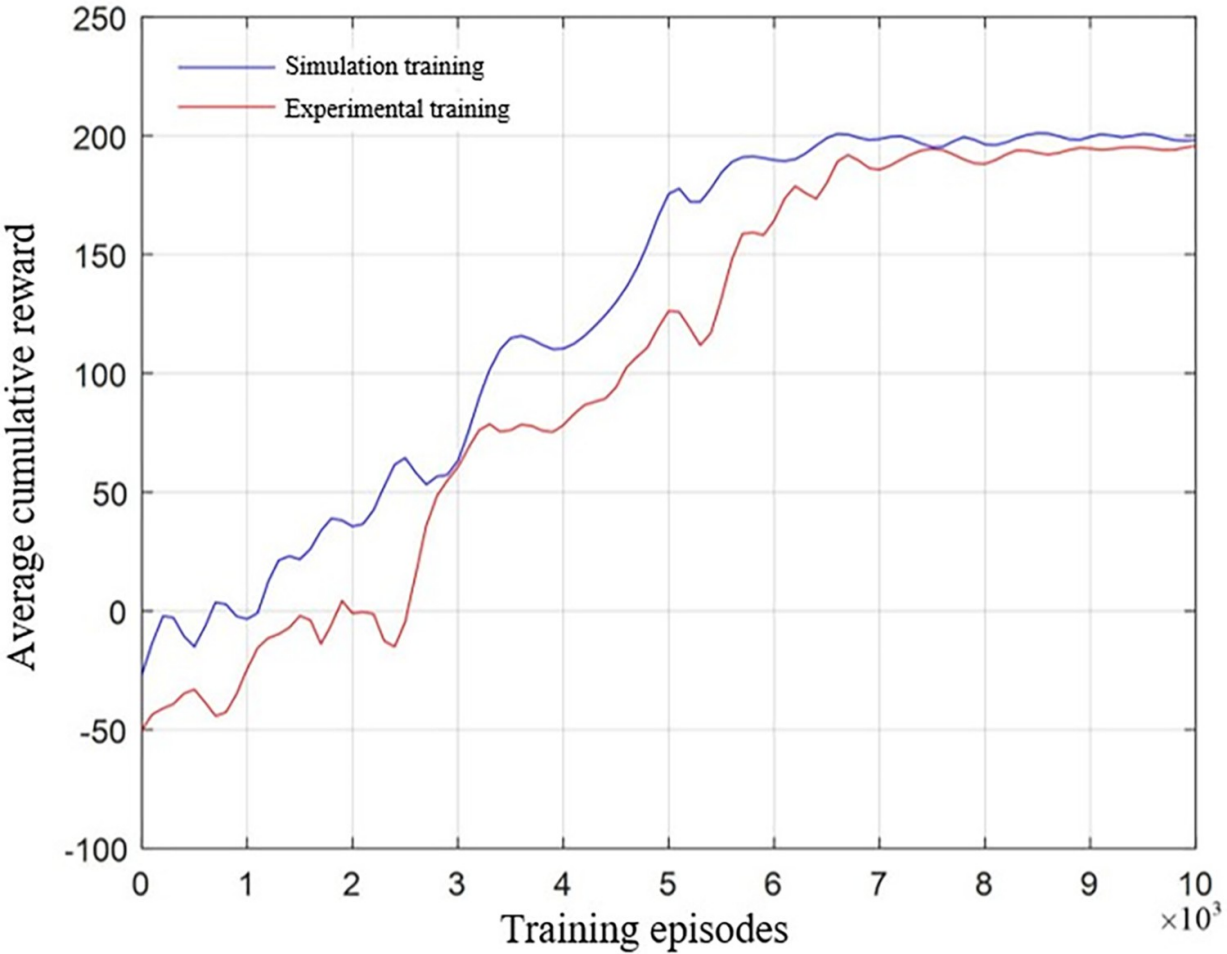
Average reward curves of experimental and simulated training.

Moveit! and the control cabinet of the Kawasaki BA006N robotic arm communicate via TCP/IP protocol. The network parameters of the DE-MADDPG algorithm are shown in **Table 3**. Based on simulation experiments, convergence typically occurs around 7000 episodes in both assembly tasks. To save time and costs, the maximum number of episodes is set to 10,000. The DE-MADDPG algorithm is used to conduct 10,000 training assemblies of the square shaft-hole task under safe conditions, with results compared to those in the simulation environment, as shown in **Fig 19**.

Although there are some discrepancies between the experimental environment and the idealized simulation environment, convergence to stability is still achieved in actual training. The improvement is slow during the initial exploration phase, as the algorithm learns to adapt to the robotic arm’s action environment. Around 3000 episodes, there is a rapid increase in rewards. However, there are considerable fluctuations in stability during the later convergence phase, caused by system errors and instabilities during mechanical movement. Nonetheless, reward values stabilize around 8000 episodes, with the final average reward slightly lower than the simulation result, ending at 182.35.

### 5.3 Generalization experiment

**Fig 20:**
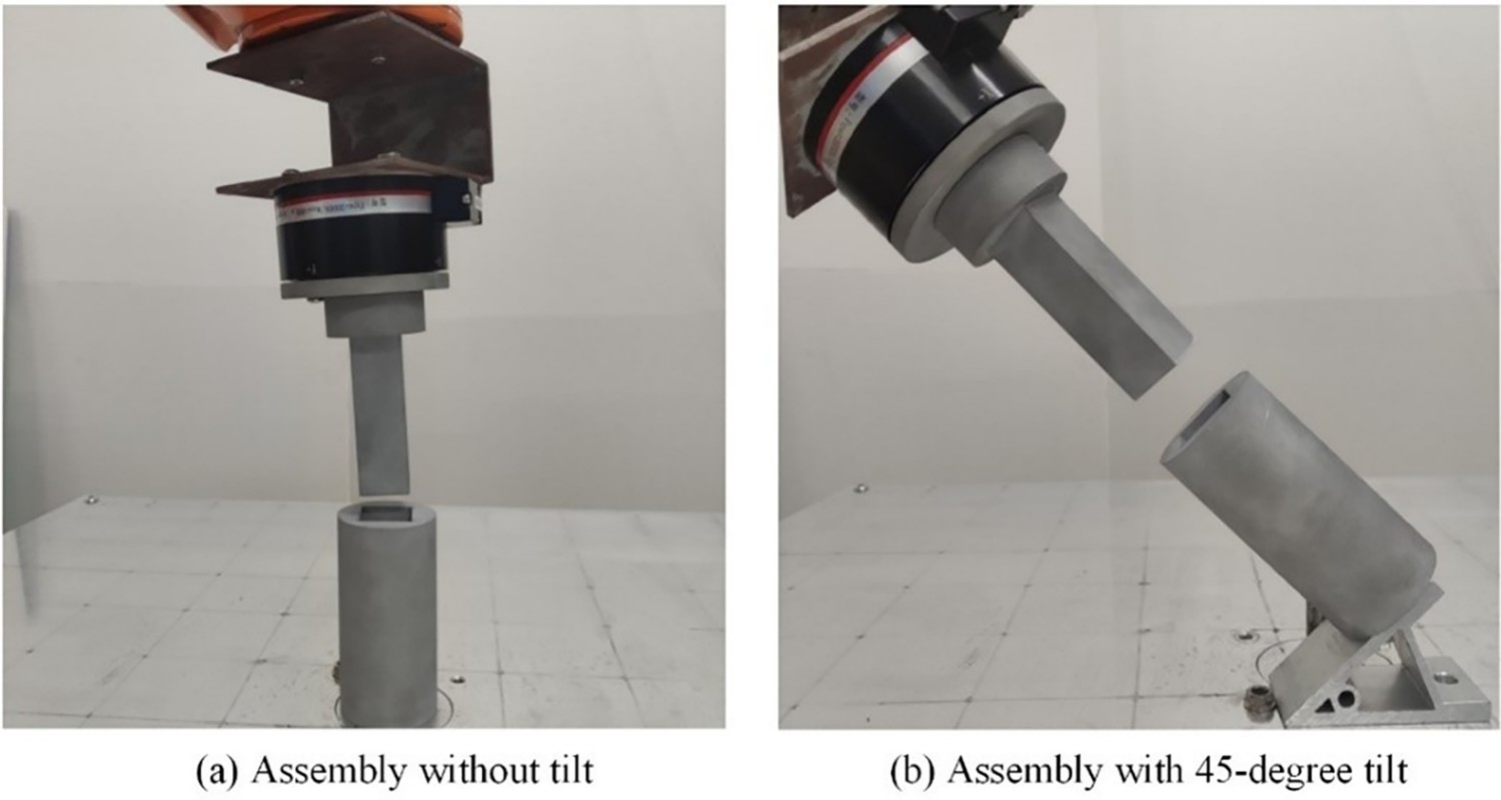
Assembly experiment object of square shaft hole.

To verify the feasibility and generalization of the training results, assembly tests of the square shaft-hole are conducted by altering the positional coordinates of the assembly hole within the workspace. The assembly training is guided by the matching of position and orientation in the algorithm’s reward function. To assess the performance of the trained model under different task conditions, four experiments are set up: (1) Experiment 1: assembly at a fixed position, (2) Experiment 2: assembly with varied positions within the working plane, (3) Experiment 3: assembly with a fixed position but with the assembly hole’s tilt angle at 45° relative to the working plane, and (4) Experiment 4: assembly with both a 45° tilt and varied position. The fixed position assembly and 45° tilt assembly are shown in **Fig 20**. Each group underwent 100 assembly trials, with the central coordinates of the contact surface after position and orientation changes calculated based on spatial relationships. The test results are summarized in **Table 6**.

**Table 6:** Comparative experiments on square shaft-hole assembly.

Experiment	DDPG		MADDPG		DE-MADDPG	
	Time per assembly(s)	Success rat%	Time per assembly(s)	Success rat%	Time per assembly(s)	Success rat%
Experiment 1	70.37	75	63.86	81	61.32	84
Experiment 2	83.14	71	75.07	76	72.89	81
Experiment 3	75.55	61	69.11	68	66.74	73
Experiment 4	92.66	52	83.69	60	79.24	66

Comparing experiments 1 and 2, the change in position leads to an altered motion trajectory, which results in a longer average assembly time, increasing from 61.32 seconds to 72.89 seconds, though the success rate remains above 80%. Between experiments 1 and 3, where only the assembly hole’s orientation is altered, the average time increased by only 5.42 seconds, but the success rate dropped from 84% to 73%. Comparing experiment 4 with experiments 2 and 3, it is evident that positional changes primarily increase assembly time, while orientation changes mainly affect the success rate. This experiment demonstrates the feasibility of using the DE-MADDPG algorithm to divide a single robotic arm into two intelligent agents controlling position and orientation for assembly tasks. Additionally, by varying the assembly hole’s position and orientation, the generalization capability of the algorithm in complex environments is validated. Across the four experiments, DE-MADDPG shows a 3%, 5%, 5%, and 6% higher success rate compared to MADDPG, and a 9%, 10%, 12%, and 14% higher success rate compared to DDPG. The results indicate that as the assembly difficulty increases, the accuracy advantage of the proposed DE-MADDPG method becomes more significant. Additionally, the average assembly time for DE-MADDPG across all four experiments is lower than that of the other two methods.

## 6. Conclusion

This paper addresses the complex assembly problem of shaft-hole structures, focusing on square shaft-hole assemblies with the Kawasaki BA006N robotic arm as the primary control entity. The research encompasses an analysis of the assembly task, the kinematics of the robotic arm, the design of a multi-agent deep reinforcement learning framework, and the formulation of multi-agent reward functions. Both simulation and real-world experiments are conducted for validation and testing. The main findings of the study are as follows: (1) The reward function for the DE-MADDPG algorithm is tailored to the assembly task process and consists of a global reward function and a local reward function. The global reward function includes a main reward function, a collision penalty function, and a process penalty function. The local rewards are divided into a position reward function, represented by Euclidean distance, and an orientation reward function, represented by quaternions. (2) A joint simulation assembly platform integrating Gazebo and Moveit! is developed within ROS. This platform is used to simulate the assembly of circular and square shaft-holes using the DE-MADDPG algorithm. The simulation results demonstrated that dividing the first three joints and the last three joints of the robotic arm into multiple agents enhances adaptability. (3) Based on the simulation results, the Kawasaki BA006N robotic arm is equipped with force/torque sensors to detect collisions, and a square shaft-hole experimental training rig is constructed. The training results show convergence consistent with the DE-MADDPG simulation outcomes.

Despite the progress made in developing a shaft-hole assembly strategy based on the DE-MADDPG algorithm, several limitations and areas for improvement remain, which can be further explored in future research: (1) The training of robotic arms in real-world environments is costly. Future research could explore the use of transfer learning or meta-learning to leverage simulation-trained models, thereby improving training efficiency. (2) The current study primarily focused on the assembly of square shaft-holes. Future experiments could validate and compare the assembly of shaft-holes with different shapes and sizes. Additionally, the testing phase only included validation at a fixed angle. Conducting assembly experiments at various angles could help assess the model’s generalization capabilities. (3) Further optimization of the reward functions and assembly tasks could enhance the system’s adaptability across different assembly environments and improve assembly success rates.
